# Development and Applications of Electrochemical Surface Plasmon Resonance (EC-SPR)-Based Sensors: A Review

**DOI:** 10.1021/acs.analchem.5c07065

**Published:** 2026-03-09

**Authors:** Jomar Sales Vasconcelos, Nazaré do Socorro Lemos Silva Vasconcelos, Cícero Wellington Brito Bezerra, Antonio Marcus Nogueira Lima

**Affiliations:** † Department of Electrical and Electronic Engineering, Federal Institute of Maranhão, Av. Getúlio Vargas 4, 65030-005 Monte Castelo, MA, Brazil; ‡ Department of Chemistry, Federal Institute of Maranhão, Av. Getúlio Vargas 4, 65030-005 Monte Castelo, MA, Brazil; ¶ Department of Chemistry, Federal University of Maranhão (UFMA), Avenida dos Portugueses 1966, 65080-805 São Luís, MA, Brazil; § Department of Electrical Engineering, Federal University of Campina Grande (UFCG), Rua Aprígio Veloso 882, Bairro Universitário, 58429-900 Campina Grande, PB, Brazil

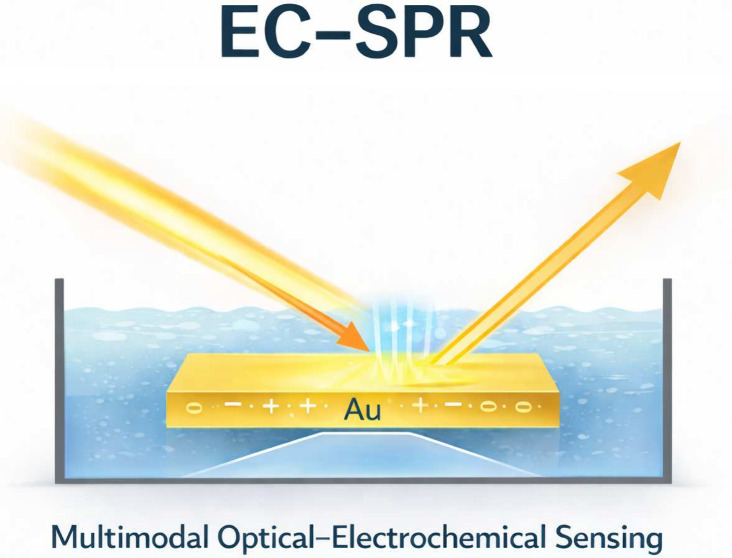

## Introduction

### General Contextualization

Sensors are devices capable of detecting changes in a system’s physical, chemical, or biological properties and converting them into measurable signals, usually electrical. These signals can then be analyzed, processed, and utilized for decision-making in various fields, including clinical diagnostics, industrial process control, environmental monitoring, and food safety. In general, a sensor consists of three main components: (i) a receptor (or sensing) element, which interacts with the analyte; (ii) a transducer, which converts this interaction into a detectable signal; and (iii) a readout system, responsible for interpreting the generated signal.^1–3^

Figure S1 schematically represents the general architecture of a sensor, emphasizing how chemical or biological recognition is transformed into measurable electrical or optical responses.

The evolution of sensor technology is rooted in the fundamental discoveries of electricity and electrochemistry, from the pioneering studies of Galvani and Volta, who demonstrated the relationship between electrical phenomena and material interfaces,^4,5^ to the development of electrochemical principles by Faraday and Nernst.^6,7^ These milestones provided the theoretical foundation for modern analytical devices capable of transforming physicochemical phenomena into measurable electrical outputs.

Among the most relevant sensors currently available are electrochemical sensors and surface plasmon resonance (SPR)-based sensors. Both are widely used due to their high sensitivity, selectivity, and applicability across different contexts, including biomedicine, the chemical industry, and environmental monitoring.

In recent years, advances in nanofabrication, conducting polymers, and AI-assisted signal processing have significantly enhanced sensor performance, enabling the development of hybrid systems that integrate various transduction principles.^2,3,8–11^

### Electrochemical Sensors

Electrochemical sensors convert chemical interactions into electrical signals and are widely used in biomedical diagnostics, environmental control, and the food industry.^12–14^ Nanomaterials, such as graphene, carbon nanotubes, and metallic nanoparticles, significantly enhance their performance, thereby improving sensitivity and selectivity. Moreover, surface modification techniques enable the detection of analytes at ultralow concentrations.^15,16^

Electrochemical sensors can be divided into three main categories: amperometric, potentiometric, and impedimetric, each one based on distinct electrochemical phenomena related to electron transfer, potential variation, or interfacial impedance.^1,2,17^ These processes are typically monitored through configurations that employ working, reference, and counter electrodes (see Figure S2), where redox reactions at the working electrode surface play a central role in signal generation.^3,15,18^ The integration of nanostructured materials with electroactive films has enabled high selectivity and stability, especially for biosensors that detect specific biomolecules through redox reactions or surface adsorption.^16,19–23^

A clearer understanding of electrochemical sensing requires describing how electrical signals originate at the electrode–solution interface and how they evolve in response to changes in analyte concentration. In systems involving a reversible redox couple O + *n*e^–^ ⇄ R, the interfacial faradaic current arises from electron-transfer kinetics and is commonly represented by the Butler–Volmer expression:

1 $$j=j_{0}\left[\exp\left(\frac{\alpha nF\eta }{RT}\right)-\exp\left(-\frac{\left(1-\alpha \right)nF\eta }{RT}\right)\right]$$

where *j*_0_ is the exchange current density, α is the charge-transfer coefficient, and η = *E* – *E*_eq_ is the overpotential applied to the interface.

Variations in the interfacial environment, such as adsorption, surface fouling, catalytic enhancement, or changes in electrolyte composition, modify these kinetic parameters and consequently reshape the recorded current–voltage profile. Under conditions of small-amplitude perturbation, the same interface can be modeled through an equivalent circuit formalism, typically described by the Randles representation:

2 $$Z\left(\omega \right)=R_{s}+\frac{1}{\frac{1}{R_{\mathrm{ct}}}+i\omega C_{\mathrm{dl}}}$$

where *R*_s_ is the solution resistance, *R*_ct_ is the charge-transfer resistance, and *C*_dl_ is the double-layer capacitance.

Shifts in ionic strength, analyte binding, or surface modification processes induce measurable variations in these parameters, resulting in changes in Nyquist semicircle dimensions and in the overall frequency-dependent impedance behavior. Such relationships form the theoretical foundation for amperometric, potentiometric, and impedimetric detection schemes widely employed in modern electrochemical sensors.

### Surface Plasmon Resonance (SPR) Sensors

SPR sensors detect changes in the refractive index of metallic films, allowing for real-time monitoring without the use of labeling agents.^3,9,10^ Their applications include biomolecular interaction studies (e.g., antigen–antibody, protein–DNA), drug development, and the analysis of environmental contaminants.^3,10,11,24,25^ In addition to allowing detailed kinetic characterization of molecular interactions, SPR sensors exhibit high precision and can detect interactions at extremely low concentrations.^3,10,11^

The physical principle of SPR is based on the excitation of collective oscillations of conduction electrons (surface plasmons) at the interface between a metal (typically gold or silver) and a dielectric medium, under conditions of resonance defined by the incident angle of polarized light.^3,10,26^ Variations in the refractive index near the metal surface alter this resonance condition, which is detected as a change in the reflected light intensity or angle (see Figure 1).^2,3,10,11^

**Figure 1:**
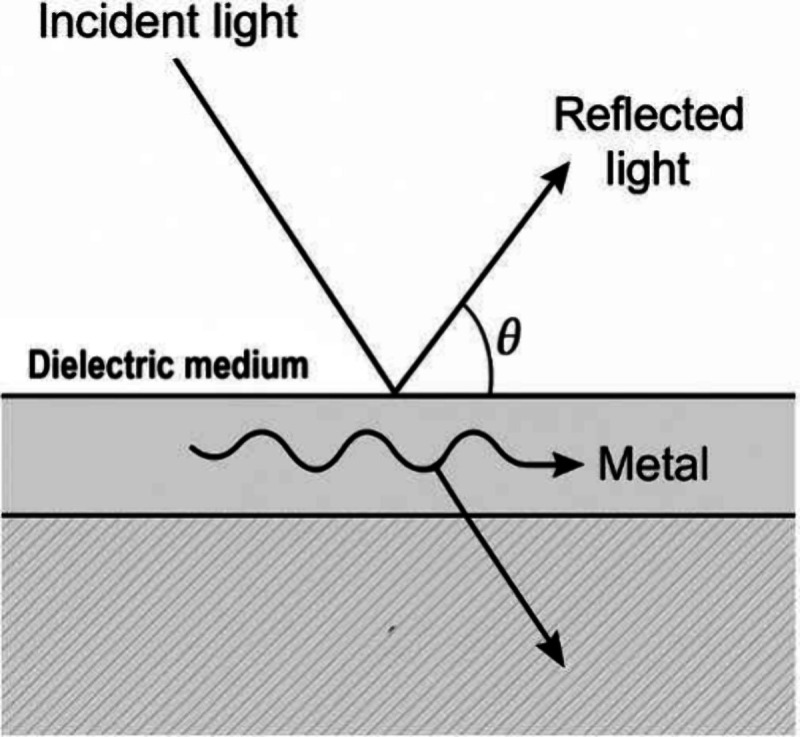
Schematic representation of the surface plasmon resonance (SPR) phenomenon.

Recent advances such as localized SPR (LSPR), SPR imaging (SPRi), and nanostructured metallic films have extended the method’s sensitivity to femtomolar levels.^3,8,27,28^ Moreover, the integration of microfluidics and miniaturized setups has expanded the applications of SPR to biosensors, environmental analysis, and point-of-care diagnostics, aspects central to the present review.^3,8,9,29,30^

To describe SPR quantitatively, it is useful to consider the momentum-matching condition that governs plasmon excitation. In the classical Kretschmann configuration, a p-polarized beam incident through a prism of refractive index *n*_p_ provides an in-plane wavevector given by

3 $$k_{x}=\frac{2\pi n_{p}}{\lambda }\;\sin\theta$$

where *k*_*x*_ is the in-plane component of the incident wavevector, λ is the incident wavelength, *n*_p_ is the refractive index of the prism, and θ is the angle of incidence.

Surface plasmons propagating at the metal–dielectric interface exhibit a characteristic wavevector determined by the dielectric constants of the metal and the sensing medium:

4 $$k_{SP}=\frac{2\pi }{\lambda }\sqrt{\frac{\varepsilon_{m}\varepsilon_{s}}{\varepsilon_{m}+\varepsilon_{s}}}$$

where *k*_SP_ is the surface plasmon wavevector, ε_m_ is the dielectric constant of the metal film, and ε_*s*_ is the dielectric constant (or refractive index squared) of the sensing medium.

SPR occurs when the coupling condition *k*_*x*_ = *k*_SP_ is satisfied, producing a sharp minimum in the reflected light intensity. Small variations in the refractive index *n*_s_ near the metal surface shift the resonance angle, which can be described to first order by

5 $$\Delta \theta_{res}=S\Delta n_{s}$$

where Δθ_res_ is the change in resonance angle, *n*_s_ is the refractive index of the sensing medium, and *S* is the angular sensitivity of the SPR configuration.

These relationships form the physical basis of SPR sensing: any process that modifies the optical properties of the interfacial region, such as biomolecular adsorption, interfacial film growth, or changes in buffer composition, produces a measurable shift in reflectivity or resonance angle.

### Integration of Electrochemical and SPR Sensors

Combining electrochemical sensors with SPR (EC-SPR) has been explored to enhance analytical sensitivity and specificity.^18,31,32^ This approach enables the simultaneous monitoring of electrochemical and optical processes, combining the speed of electrochemical sensing with the molecular precision of SPR characterization.^18,30,31^ Applications include hybrid biosensors for early disease detection and environmental monitoring.^3,16,30^ In EC-SPR systems, the potential applied to the working electrode modulates the surface charge density, thereby altering the local refractive index and affecting the plasmonic resonance condition.^18,31,33^ This coupling between the electrochemical and optical domains provides real-time information about adsorption, redox kinetics, and film formation, surpassing the capabilities of each technique individually (see Figure 2).^18,31,34–36^

**Figure 2:**
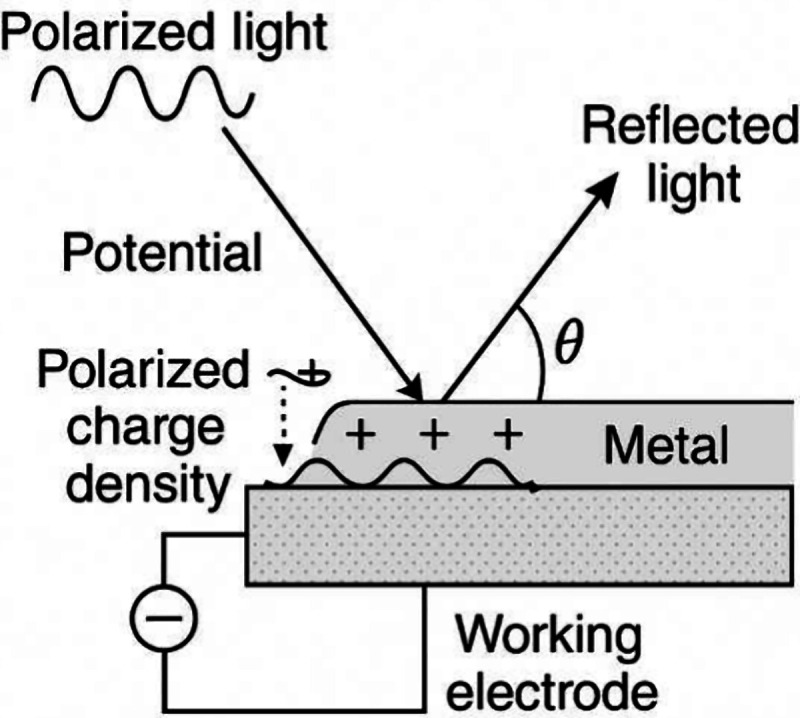
Schematic representation of the electrochemical surface plasmon resonance (EC-SPR) principle.

The convergence of EC and SPR enables the direct correlation of charge-transfer phenomena with optical responses, allowing, for example, the detection of biochemical reactions or pollutant adsorption with higher precision.^18,30–32^ This hybridization represents one of the most significant trends in modern analytical instrumentation, combining the quantitative power of electrochemistry with the molecular specificity of plasmonic sensing.^3,18,30,31^

### Coupling Mechanism in EC-SPR

The coupling between electrochemical excitation and plasmonic response arises from the sensitivity of surface plasmons to changes in charge density, refractive index, and structural organization at the metal–electrolyte interface. When an external potential is applied to the gold working electrode in an EC-SPR system, the interfacial charge distribution is modulated according to classical electrochemical principles. This modulation alters the structure of the electrical double layer (EDL), changing both the surface charge density σ and the local refractive index *n*(*z*) within the evanescent field penetration depth (typically 150–300 nm). For a metal–electrolyte interface, the change in surface charge induced by an applied potential *E* follows

6 $$\sigma =C_{\mathrm{dl}}\left(E-E_{\mathrm{pzc}}\right)$$

where *C*_dl_ is the double-layer capacitance and *E*_pzc_ is the potential of zero charge.

Variations in σ modify the ionic distribution described by the Poisson–Boltzmann equation, producing a refractive-index shift:

7 $$\Delta n\left(z\right)=f_{1}\left(\Delta \rho ,\Delta \varepsilon \right)$$

This refractive index perturbation directly affects the SPR resonance condition defined by the Fresnel formalism:

8 $$\theta_{\mathrm{SPR}}=f_{2}\left(n_{\mathrm{metal}},n_{\mathrm{interface}},n_{\mathrm{medium}}\right)$$

yielding a measurable angular or wavelength shift Δθ_SPR_ proportional to Δ*n*.

Consequently, any redox process, adsorption/desorption event, electropolymerization step, or mass redistribution within the interfacial region results in simultaneous electrochemical and plasmonic signatures.

In redox-active films, changes in oxidation state produce additional contributions to the coupling mechanism. Modulation of electronic density in conducting polymers or transition metal complexes alters the complex dielectric constant of the film:

9 $$\varepsilon =\varepsilon^{\prime }+i\varepsilon^{\prime \prime }$$

yielding a measurable angular or wavelength shift Δθ_SPR_ proportional to Δ*n*, thereby affecting plasmon propagation and increasing the optical contrast in EC-SPR measurements. Similarly, electropolymerization and electrodeposition introduce dynamic changes in film thickness and morphology, which modify both the optical path and the electron density at the interface. Overall, EC-SPR coupling results from the superposition of electrostatic modulation of the EDL, redox-induced changes in dielectric properties and structural or mass variations occurring within the penetration depth of the evanescent plasmonic field. This multicomponent mechanism is the foundation for simultaneous optical and electrochemical interrogation, enabling molecular-level insights into interfacial processes that cannot be accessed using either technique alone.

### Objective of the Review

This review examines the advances in EC-SPR technology, highlighting its impact on miniaturization, sensitivity, and emerging applications. Integrating these techniques has driven research in biomedicine, the development of portable sensors, and the real-time detection of pathogens. The review also discusses future trends, including nanomaterials, conducting polymers, and new transduction mechanisms, as well as the challenges to be addressed, such as enhancing the robustness and reproducibility of sensors. By providing a structured and critical overview, this review aims to clarify the mechanisms underlying EC-SPR convergence, identify limitations in current research, and propose directions for more robust, reproducible, and application-oriented sensor designs.

## Historical Background and Development of Electrochemistry and Surface Plasmon Resonance

### Development of Electrochemistry (EC)

Electrochemistry originated in the 18th century with the pioneering experiments of Luigi Galvani and Alessandro Volta. Galvani observed what he termed “animal electricity”,^4^ while Volta demonstrated that electric current could be generated through contact between dissimilar metals, leading to the invention of the voltaic pile in 1800.^5^ This discovery catalyzed further studies, including the electrolysis of water (Nicholson and Carlisle),^36^ the synthesis of elements such as sodium, potassium, and calcium by Humphry Davy,^37^ and the formulation of the electrolysis laws by his assistant Michael Faraday, who also introduced key electrochemical concepts such as anode, cathode, and ions.^6^

Subsequently, Svante Arrhenius proposed the electrolytic dissociation theory to explain the conductivity of electrolytes, suggesting that molecules dissociate spontaneously into ions when dissolved in an aqueous solution, even in the absence of an external electric current.^38^ Wilhelm Ostwald’s dilution law provided a means to calculate equilibrium constants, offering experimental support for Arrhenius’ theory.^39^ Applying thermodynamic principles to ionic behavior, Walther Nernst established the relationship between electrochemical potential and ionic concentration in solution,^7^ which was later refined and historically reviewed by Scholz^40^ and Bard and Faulkner.^41^

However, Arrhenius’ theory exhibited limitations in describing the behavior of strong electrolytes, which appeared to dissociate completely yet displayed variations in conductivity with concentration. To explain this phenomenon, Friedrich Kohlrausch demonstrated that the molar conductivity of strong electrolytes decreases with increasing concentration. Later, in 1923, Debye and Hückel introduced a theory based on electrostatic interactions between ions, proposing the concept of an ionic atmosphere to correct Arrhenius’ model for concentrated solutions. These contributions laid the foundations of modern electrochemistry by providing a more accurate understanding of ionic behavior in aqueous systems.^41–44^

By the late 19th and early 20th centuries, electrochemistry had become critical for industrial applications, such as metal electrorefining, electroplating, and aluminum production via the Hall–Héroult process.^44–46^ Techniques such as voltammetry and polarography (Heyrovský) enabled the quantitative analysis of electroactive species.^47^ In the 20th century, advancements in potentiometry, conductometry, and coulometry allowed for more precise measurements and the automation of electrochemical processes.^41^ These innovations significantly advanced fields such as water treatment, chemical synthesis, and electrochemical machining.^41,46^

Thus, the maturation of electrochemistry throughout the 20th century not only established its theoretical and industrial foundation but also paved the way for the emergence of modern analytical and biosensing technologies, which later converged with optical detection methods such as SPR.

### Industrial Electrochemistry

Industrial electrochemistry experienced significant expansion at the end of the 19th century, driven by the growing demand for electrical energy and the increasing adoption of large-scale manufacturing processes. The chlor-alkali process became one of the most important electrochemical processes, employing diaphragm, mercury, and membrane cells to produce chlorine, hydrogen, and sodium hydroxide.^48^ Copper electrorefining was a key process for purifying the metal for electrical applications.^29^ The development of dimensionally stable anodes (DSAs) by Henri Beer reduced energy consumption and improved the efficiency of chlor-alkali cells.^49^ Introducing ion-exchange membranes, such as Nafion, revolutionized ion separation in industrial electrochemical systems.^50^ These industrial breakthroughs also accelerated the development of electrochemical sensors, as principles of electrode stability, current efficiency, and ionic transport began to be translated into analytical devices.^2,3^ The same technological foundations that improved large-scale electrolyzers later enabled the miniaturization and precision control required for sensing systems.^17,50^

### Simulations and Mathematical Modeling in Electrochemistry

Computational simulations have become essential for modeling redox reactions, charge transfer, and ion transport. Techniques such as molecular dynamics (MD) and density functional theory (DFT) enable the atomic-level understanding of electrochemical interactions.^51^ Mathematical models based on Fick’s laws (species diffusion), the Nernst–Planck equation (ion transport), the Butler–Volmer equation (charge transfer kinetics), and Poisson’s equation (electric potential distribution) are widely used to optimize batteries, sensors, and advanced electrochemical systems.^7,17,40^

Electrochemical simulations are typically based on fundamental equations that describe mass transfer, surface reactions, and charge transport. Some of the key equations used are shown below.

Fick’s law describes the diffusion of electrochemical species in the electrolyte:

10 $$\frac{\partial C_{i}}{\partial t}=D_{i}\nabla^{2}C_{i}$$

where *C*_*i*_ is the concentration of species *i*, *D*_*i*_ is the diffusion coefficient, and *t* is time. This equation is fundamental for modeling species distribution in batteries and fuel cells.

The Nernst–Planck equation accounts for the influence of electric fields on the transport of charged species:

11 $$J_{i}=-D_{i}\nabla C_{i}-\frac{z_{i}FD_{i}}{RT}C_{i}\nabla \phi +C_{i}v$$

where *J*_*i*_ is the flux of species *i*, *z*_*i*_ is its charge, *F* is the Faraday constant, *R* is the gas constant, *T* is the temperature, *v* is the fluid velocity, and ϕ is the electric potential.

The Butler–Volmer equation describes the charge transfer kinetics at the electrode–electrolyte interface:

12 $$j=j_{0}\left[\exp\left(\frac{\alpha_{a}nF\eta }{RT}\right)-\exp\left(-\frac{\alpha_{c}nF\eta }{RT}\right)\right]$$

where *j*_0_ is the exchange current density, α is the charge-transfer coefficient, and η = *E* – *E*_eq_ is the overpotential applied to the interface.

Poisson’s equation determines the distribution of the electric potential:

13 $$\nabla^{2}\phi =-\frac{\rho }{\epsilon }$$

where ρ is the charge density and ϵ is the permittivity of the medium. This equation is critical for modeling the electric field around electrodes.

In systems where convective flow is significant, such as in fuel cells, the Stokes equation is used to model fluid motion:

14 $$\rho \left(\frac{\partial v}{\partial t}+v\nabla v\right)=-\nabla p+\mu \nabla^{2}v+f$$

where *v* is the fluid velocity, *p* is the pressure, μ is the dynamic viscosity, and *f* represents body forces such as gravity.

Figure S3 schematically summarizes the relationships among these equations, showing how diffusion, migration, and convection govern mass transport, which in turn couples to charge-transfer kinetics and electric potential distributions. This conceptual map illustrates the connection between mathematical modeling and the relationship between microscopic ion motion and macroscopic electrochemical performance.

### Advanced Applications

Electrochemistry plays a pivotal role in the development of lithium-ion batteries, fuel cells, and electrochemical sensors. Simulations are used to predict material degradation, catalytic efficiency, and the behavior of protective films. Electrochemical sensors, optimized through computational modeling, are widely used for contaminant detection, biomedical monitoring, and industrial process control.^2^ Continuous advancements in electrochemical engineering are strengthening its role in renewable energy, biotechnology, and sustainable chemistry, making it an indispensable tool for technological innovation.^52^ The synergy between experimental electrochemistry and computational modeling has become central to innovation, enabling predictive sensor design and optimization of electrode materials under real-world conditions. This cross-domain integration forms the foundation for the hybrid EC-SPR systems discussed in the next section.

### Development of Surface Plasmon Resonance (SPR)

SPR is a fundamental technique for analyzing surfaces and interfaces. Its applications range from biomolecular interactions and sensor development to the characterization of novel materials. Although its theoretical foundations were established in the early 20th century, practical applications only emerged during the 1960s and 1970s.^3,53–55^

Figure S4 schematically represents the principle of SPR excitation in the Kretschmann configuration, illustrating how a p-polarized light beam couples with surface plasmons at the metal–dielectric interface under specific resonance conditions.

### Fundamentals of SPR

SPR occurs when incident light at a metal–dielectric interface excites surface plasmon waves, decreasing reflected light intensity. This phenomenon is sensitive to changes in the interface’s refractive index, enabling the detection of precise molecular interactions. Noble metals such as gold and silver are commonly used due to their favorable optical properties. Otto, Kretschmann, and Raether’s pioneering work in the 1960s and 1970s established the experimental basis of SPR, employing prism-based coupling to study metallic surfaces. The Kretschmann–Raether configuration remains widely used for molecular adsorption detection and thin-film analysis.^3,53–55^

The physical mechanism is governed by the interaction between the evanescent electromagnetic field and free electrons at the metal surface, generating surface plasmons that are highly sensitive to refractive-index perturbations. This optical resonance forms the foundation for label-free biosensing and characterization of thin films.^3,54–56^

### Simulations and Modeling of SPR

Simulations based on Maxwell’s theory are essential for predicting and optimizing SPR responses. They enable the modeling of parameters such as incidence angle, light polarization, and optical properties of materials. These models are fundamental for developing more sensitive sensors and precisely calibrating experiments.^3,10,11,56^ The main mathematical models employed include the following:

- Maxwell’s equations for describing the propagation of electromagnetic waves;
- the Fresnel equations for calculating the light reflection coefficient;
- the Drude model for describing the dielectric response of metals.

The surface plasmon resonance condition defines the coupling between incident light and surface plasmons. These models are crucial for quantitative analysis and sensor optimization, enabling the prediction of signal variations resulting from environmental changes or modifications in surface composition.^3,10,11,56^ SPR is governed by fundamental equations that describe the propagation and interactions of electromagnetic waves with metallic surfaces. The key equations and models used to describe the SPR phenomenon are given below.

Maxwell’s equations, the theoretical basis for modeling electromagnetic wave propagation across different media, are expressed as

15 $$\nabla \cdot E=\frac{\rho }{\epsilon_{0}},,\nabla \cdot B=0$$

16 $$\nabla \times E=-\frac{\partial B}{\partial t},,\nabla \times B=-\mu_{0}J+\mu_{0}\epsilon_{0}\frac{\partial E}{\partial t}$$

where **E** is the electric field, **B** is the magnetic field, ρ is the charge density, **J** is the current density, ϵ_0_ is the vacuum permittivity, and μ_0_ is the vacuum permeability.

By assuming time-harmonic electromagnetic fields, Maxwell’s equations can be reduced to the Helmholtz equation, which is widely used to model electromagnetic wave propagation in multilayer plasmonic systems and forms the theoretical basis for analytical and numerical SPR simulations.

17 $$\nabla^{2}E+{k_{0}}^{2}\varepsilon \left(\omega \right)E=0$$

where *k*_0_ = ω/*c* is the wavevector in vacuum and ε­(ω) is the frequency-dependent dielectric function of the medium, commonly described for metals using the Drude model.

The SPR resonance condition occurs when the wavevector of the incident light matches that of the surface plasmon, described as

18 $$k_{\mathrm{SP}}=k_{0}\sqrt{\frac{\varepsilon_{m}\varepsilon_{d}}{\varepsilon_{m}+\varepsilon_{d}}}$$

where *k*_SP_ is the surface plasmon wavevector modulus, *k*_0_ is the wavevector of light in vacuum, and ε_m_ and ε_d_ are the dielectric constants of the metal and dielectric, respectively.

Fresnel’s equation is used to calculate the reflection coefficient *R*(θ) as a function of the incidence angle θ, which is key to determining the SPR resonance angle:

19 $$R\left(\theta \right)={\left|\frac{n_{1}\cos\theta -n_{2}\sqrt{1-{\left(\frac{n_{1}}{n_{2}}\;\sin\theta \right)}^{2}}}{n_{1}\cos\theta +n_{2}\sqrt{1-{\left(\frac{n_{1}}{n_{2}}\;\sin\theta \right)}^{2}}}\right|}^{2}$$

where *n*_1_ and *n*_2_ are the refractive indices of the adjacent media (incident and dielectric).

The Drude model is frequently used to describe the metal’s dielectric function, which is crucial for determining the SPR response:

20 $$\varepsilon_{m}\left(\omega \right)=\varepsilon_{\infty }-\frac{{\omega_{p}}^{2}}{\omega^{2}+i\gamma \omega }$$

where *ε*_*∞*_ is the high-frequency dielectric constant, ω_*p*_ is the plasma frequency, ω is the angular frequency of the incident light, and γ is the damping constant.

The relationship between the change in resonance angle δθ and the refractive index variation ∂*n* can describe the sensitivity of an SPR sensor:

21 $$S_{\mathrm{SPR}}=\frac{\partial \theta }{\partial n}$$

This parameter is crucial for assessing a sensor’s ability to detect subtle changes in the dielectric environment near the metallic surface.

The combination of these equations enables the accurate modeling of reflectivity curves and field distributions in multilayer systems, which is crucial for designing nanostructured plasmonic chips.

### Expansion into Biotechnology and Life Sciences

Since the 1980s, SPR has revolutionized biomedical and biotechnological research by enabling label-free detection of biomolecular interactions. SPR-based biosensors have been developed to analyze antigen–antibody, DNA, and protein interactions, allowing for detailed kinetic analysis of molecular binding events.^3,55^

Additionally, SPR has become essential in drug discovery, enabling high-throughput screening of compound libraries for potential therapeutic candidates. The technique is also used in evaluating the biocompatibility of materials and immunological responses, thereby expanding its applications in healthcare and biomedical research.^57–60^

The convergence of SPR with electrochemical platforms (EC-SPR) has further extended its role in medical diagnostics, enabling real-time correlation between optical and electrochemical signals for improved disease biomarker detection and molecular affinity studies.^16,61^

### Technological Advances and Enhanced Sensitivity

During the 1990s and 2000s, SPR advanced significantly through improvements in optics, electronics, and materials science. Introducing metallic nanoparticles enabled signal amplification, increasing sensitivity to levels suitable for detecting interactions at extremely low concentrations.

The development of miniaturized sensors and automated platforms has expanded the applications of SPR in clinical diagnostics, environmental monitoring, and cell–cell interaction studies. Modern SPR sensors can highly detect pathogens, disease biomarkers, and chemical compounds, becoming essential tools for rapid and reliable analysis.^3^

Surface nanostructuring and localized SPR (LSPR) have enabled single-particle detection and multiplexed analysis, while microfluidic integration has improved sample handling and real-time quantification.^3^

### Integration with Other Techniques and Hybrid Technologies

The integration of SPR with other analytical techniques has broadened its capabilities. Combining SPR with electrochemistry (EC-SPR) enables simultaneous detection of optical and electrochemical changes, with applications in electrodeposition, charge transfer studies, and hybrid biosensor development.^3,61^

Other combinations, such as SPR with Raman or infrared spectroscopy (IR-SPR), offer detailed characterization of surface and molecular interactions. These hybrid approaches enhance the understanding of surface chemistry phenomena, support the development of novel materials, and contribute to advancements in nanotechnology.^8,62^

The continuous evolution of hybrid plasmonic technologies marks the transition from conventional optical sensing to multifunctional analytical systems, a key focus of the section describing the instrumentation and cell configuration of EC-SPR systems.

### Integration of Electrochemistry (EC) and Surface Plasmon Resonance (SPR): EC-SPR

The integration of surface plasmon resonance (SPR) and electrochemistry (EC) has emerged as an innovative approach for analyzing surfaces and interfaces, combining electrochemical sensitivity with real-time optical detection. EC-SPR enables simultaneous monitoring of redox processes, molecular adsorption, and structural changes on electrodes, establishing itself as a powerful tool in various fields, including nanotechnology, biotechnology, and materials science.^18^

Figure 3 schematically illustrates the principle of EC-SPR, showing how an electrochemical cell coupled to a plasmonic film allows the applied potential to modulate the local refractive index, thus correlating optical and electrochemical signals in real time.

**Figure 3:**
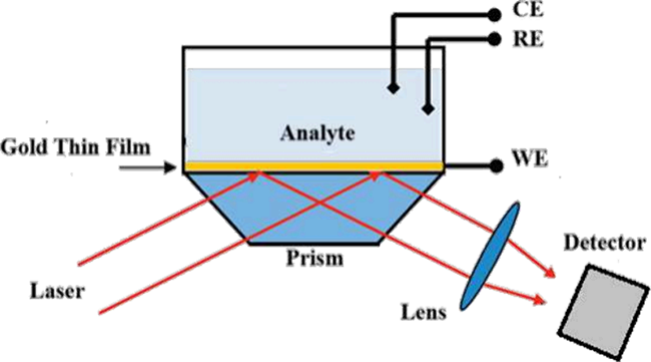
Principle of EC-SPR coupling between applied potential and plasmonic response. Shown is a schematic representation of an EC-SPR configuration, redrawn by the authors based on established designs reported in the literature.

### Fundamentals and Motivations of EC-SPR

SPR is a phenomenon based on the excitation of surface plasmons—coherent oscillations of free electrons—at the interface between a noble metal, such as gold or silver, and a dielectric medium. These surface plasmons are highly sensitive to changes in the refractive index at the metal–dielectric interface, making SPR an exceptionally powerful tool for detecting molecular interactions and environmental changes at surfaces.

Electrochemical (EC) techniques, including voltammetry and chronoamperometry, are widely used for their high sensitivity in probing redox reactions. However, these methods often lack the ability to provide detailed structural information about the species or processes occurring at the interface.

The integration of electrochemistry with SPR (EC-SPR) was developed to address these limitations. By combining these two techniques, researchers can simultaneously acquire both optical and electrochemical data, significantly enhancing the sensitivity and specificity for characterizing interfacial processes. In EC-SPR, an applied potential to the working electrode induces charge redistribution at the metal surface, which alters the local dielectric environment and, consequently, the resonance condition of the surface plasmons. This results in an optical shift (such as a change in resonance angle Δθ or reflectivity Δ*R*) that directly reflects the electrochemical events at the interface.

This coupling enables the distinction between various interfacial phenomena, such as electron transfer, adsorption, and morphological changes, with nanometric precision.^18,49^

The primary motivation behind EC-SPR lies in its unique ability to directly link electron flow (monitored electrochemically) to changes in optical permittivity (monitored by SPR). This synergy bridges the gap between charge dynamics and structural phenomena at interfaces—an insight that cannot be achieved by either technique alone.^8,18^

### Convergence of Electrochemistry and Surface Plasmon Resonance (EC-SPR)

The convergence of electrochemistry (EC) and surface plasmon resonance (SPR) emerged from the need to correlate electronic charge transfer with molecular and structural changes occurring at electrode interfaces. While electrochemical methods provide quantitative information on redox kinetics and current responses, they cannot resolve the structural or dielectric variations that accompany these processes. Conversely, SPR offers nanometric sensitivity to refractive index changes but cannot distinguish the electrochemical nature of the event. EC-SPR bridges this gap by allowing the simultaneous acquisition of optical and electrochemical data, thereby enabling the direct visualization of redox-induced surface transformations in real-time. This convergence has been crucial for developing sensors capable of distinguishing between physical adsorption and chemical reactions, thereby improving selectivity, reproducibility, and the mechanistic understanding of interfacial phenomena.

### Early Developments and Initial Applications

The first EC-SPR experiments were conducted in the 1980s and 1990s, focusing on the monitoring of oxide film formation on metal electrodes and the adsorption/desorption of molecules during potential variation. These studies demonstrated that EC-SPR could provide complementary insights into the structural and kinetic aspects of electrochemical reactions.^18^ One key early application involved studying the electrochemical oxidation and thin-film growth on electrodes, allowing researchers to correlate SPR optical variations with chemical transformations occurring at the interface.^18^

During this pioneering period, researchers such as Royce, Harrington, and Kunze established protocols that integrated potentiostats with optical detection modules. These systems demonstrated that shifts in plasmonic resonance were directly proportional to changes in electron density, providing the first quantitative validation of EC-SPR coupling.^18,63^

### Technological Advancements and Improvements in EC-SPR

With the technique’s evolution, new optical systems and electrode materials were developed, including the following:

- use of variable-wavelength lasers, enhancing precision in plasmon excitation;
- development of electrodes modified with metallic nanoparticles, improving both electrochemical and optical sensitivity;
- automation of EC-SPR systems, increasing reproducibility and enabling broader application in industrial and academic laboratories.

These advances have expanded EC-SPR applications into areas such as electrocatalysis, material corrosion, and biomolecular sensing.^48,63^

Furthermore, advances in microfabrication and transparent conducting substrates (e.g., ITO, FTO, graphene) have improved charge transfer while maintaining optical coupling efficiency. The introduction of nanostructured electrodes and localized plasmonic hotspots has enhanced the detection limit to the femtomolar range.^11,18^

### Current Applications and the Impact of EC-SPR

EC-SPR has become essential for studying complex redox processes, including the following:

- electrocatalyst characterization, allowing simultaneous analysis of catalytic activity and surface changes in materials;
- electrochemical biosensors, detecting biomolecular interactions such as antigen–antibody and DNA–protein binding;
- corrosion monitoring and metal protection, aiding in the optimization of anticorrosive coatings.

Furthermore, the technique has been increasingly applied to the development of advanced sensors for environmental and health monitoring, enabling real-time detection of pathogens, biomarkers, and chemical contaminants.^64^

Recent work also demonstrates EC-SPR’s role in studying charge-transfer kinetics, ion intercalation in energy materials, and photoelectrocatalytic mechanisms. Its versatility bridges the gap between basic electrochemical research and applied diagnostics, consolidating EC-SPR as a cornerstone of modern hybrid sensing technologies.^18,31,65^

## Overview of Representative EC-SPR Studies

The scientific development of EC-SPR has been shaped by diverse contributions involving advances in materials, electrode architectures, electrochemical modulation strategies, and plasmonic configurations. To provide a coherent perspective on how these elements have evolved, this review highlights representative publications that illustrate major conceptual and technological trends in the field. These examples are organized in a comparative table to facilitate visualization of methodological progress, innovations in sensor design, and emerging application areas. Importantly, the table is not intended to be exhaustive; rather, it complements the broader discussion supported by the full set of references examined in this review.

Table 1 summarizes the most prolific authors in the field of electrochemical surface plasmon resonance (EC-SPR), identified through a bibliometric screening of the EC-SPR literature indexed in major scientific databases over the period from 2003 to 2025. The selection criterion was based exclusively on publication frequency, considering researchers with at least six peer-reviewed EC-SPR articles within this time frame. This approach highlights key contributors whose sustained research output has significantly shaped the development of EC-SPR methodologies.

**Table 1:** Most Prolific Authors in EC-SPR Research (2003–2025), Selected Based on Publication Frequency (Six or More EC-SPR Articles)

author	affiliation	number of EC-SPR Publications (2003–2025)	main research contributions	ref
Akira Baba	Niigata University, Japan	16	Electrochemical interfaces, EC-SPR spectroscopy, surface analysis, hybrid sensor development	^66^
Keizo Kato	Niigata University, Japan	16	Nanomaterials, silicon nanowire fabrication, optical–electrochemical interfaces, advanced material characterization	^67^
Kazunari Shinbo	Niigata University, Japan	9	SPR and EC-SPR instrumentation, optoelectronic device development, thin-film plasmonics.	^68^
Futao Kaneko	Niigata University, Japan	9	Conducting polymer films, dual biosensors, EC-SPR thin-film growth analysis, battery degradation studies	^69^
Sukon Phanichphant	Chiang Mai University, Thailand	7	Nanoparticle synthesis, electrochemical and optical sensing, catalyst–sensor interfaces.	^70^
Saengrawee Sriwichai	Chiang Mai University, Thailand	7	Polymers and nanomaterials, EC-SPR material characterization, sensor development.	^71^
Rigoberto C. Advincula	University of Tennessee/ORNL, USA	6	Polymer brushes, advanced coatings, electroactive materials, EC-SPR applied to soft matter.	^72^
Rapiphun Janmanee	Pibulsongkram Rajabhat University, Thailand	6	EC-SPR sensor development, polymer films, nanostructured interfaces, editorial leadership in scientific dissemination.	^73^

A notable pattern emerging from the literature is the formation of geographically cohesive research clusters, particularly in Japan, Thailand, and the United States, whose collaborative output has significantly influenced the direction and scope of EC-SPR research. Their contributions range from fundamental investigations of plasmon–charge interactions to the development of advanced biosensing strategies and electroactive thin-film platforms.

In parallel, keyword analyses reveal recurrent themes across the EC-SPR literature, including electropolymerization, redox mechanisms, conducting polymers, surface plasmons, graphene-based hybrid interfaces, and ferrocene derivatives. These topics reflect the central role of interfacial chemistry and nanostructured materials in shaping the evolution of EC-SPR methodologies.

Overall, the combination of representative publications, collaboration patterns, and recurring thematic trends provides a concise yet comprehensive view of the state of the art in EC-SPR. This synthesis establishes a foundation for discussing technical challenges, innovative solutions, and future opportunities addressed in subsequent sections of this review.

## Technological Challenges

The development of EC-SPR sensors faces several challenges that affect their accuracy, sensitivity, and applicability. One of the primary issues is background signal suppression, which compromises electrochemical stability and the propagation of plasmon waves. Two-dimensional materials, such as graphene and MoS_2_, have been explored to mitigate noise and improve signal quality.^16,74–79^

Figure S5 schematically summarizes the main technological challenges in EC-SPR systems, including noise suppression, optimization of multilayer films, and interfacial stability. The diagram illustrates how nanomaterials, such as graphene and MoS_2_, are integrated to enhance both electrochemical and plasmonic performance.

Sensitivity and selectivity are also critical, especially for detecting biomolecules and metal ions. The integration of high-resolution SPR with anodic stripping voltammetry (ASV) has demonstrated potential for precise and portable analyses, with notable applications in environmental and biomedical fields.^9,15,19,20,67,75–77,80–82^ Surface modification with self-assembled monolayers (SAMs) has shown that redox reactions alter the tilt of alkyl chains, directly affecting the sensor’s response.^83^ A persistent challenge in EC-SPR sensor development lies in achieving sufficient sensitivity without compromising film stability or selectivity. In many cases, conventional conducting polymers or self-assembled monolayers fail to generate detectable optical shifts for low analyte concentrations, motivating the search for materials and mechanisms that can amplify the plasmonic response.

Characterizing the electrochemical and optical properties of sensors is another major challenge. Accurate determination of conductivity and dielectric constants is crucial for optimizing sensor performance under varying conditions. Although advanced mathematical modeling has been applied, the complexity of materials still presents obstacles^15,82–88^

Real-time monitoring of electrochemical processes has been advanced through techniques such as EC-SPR and quartz crystal microbalance (QCM). These methodologies have been employed to analyze electroactive biofilms (EABs), investigate redox interactions in wastewater, and detect organic contaminants.^14,89^ EC-SPR has also been used to evaluate redox interactions between proteins such as cytochrome c and cytochrome c oxidase, enabling kinetic calculations and detailed in vivo analysis.^34,90^

Interference and noise in measurements continue to be recurring challenges. Difficulty in distinguishing relevant signals from background noise, particularly in the presence of ascorbic acid and urate, compromises the reliability of selective detection. The use of a two-electrode configuration has proven to be a promising solution to minimize interference, eliminating the need for reference electrodes and reducing noise sources.^91^

Controlling molecular reorganization in self-assembled monolayers (SAMs) is essential for optimizing EC-SPR sensors. The orientation of dipoles in α-helical peptides has been shown to have a significant influence on sensor response, allowing for customized surface modulation. Moreover, the covalent immobilization of anti-IgG and anti-IgM antibodies has demonstrated high stability and specificity, making it essential for advanced immunological biosensors.^92^

The structural optimization of EC-SPR sensors aims to enhance resolution and reduce noise by utilizing materials with tunable optical properties and novel deposition techniques. These advances are critical for expanding EC-SPR applications in environmental monitoring, clinical biosensors, and the analysis of complex electrochemical reactions.^51,93,94^

Integrating electrochemical and optical phenomena requires innovative approaches that combine the development of new materials, advanced characterization techniques, and sensor optimization, ensuring improved sensitivity and broader applicability of EC-SPR technology.

## Proposed Solutions

The use of multilayer films and nanomaterials with precisely controlled thickness has improved EC-SPR sensor performance. Techniques such as layer-by-layer (LbL) deposition and electropolymerization have proven effective in constructing conjugated polymer networks, optimizing redox properties and structural stability for advanced chemical and biomedical analyses. Graphene layer deposition on gold chips has also been employed to enhance electrochemical stability, sensitivity, and electrical conductivity, thereby broadening the applicability of EC-SPR sensors.^14,32,33,78–80,90,95–97^ The advancement of EC-SPR sensors has benefited from the integration of techniques such as electrochemical impedance spectroscopy (EIS), quartz crystal microbalance (QCM), and atomic force microscopy (AFM), which enhance device sensitivity and selectivity^15,22,23,63,80,81,83,93,95,96,98^

Charge-transfer complexes (CTCs) formed between polymerized *o*-tolidine and dermatan sulfate (DS) have been shown to significantly enhance the SPR signal, achieving detection limits as low as 8 nM.^97^ This improvement stems from redox-induced modulation of the refractive index within the CTC matrix, which enhances sensitivity and facilitates electrode surface regeneration. Although this strategy represents a material-specific solution rather than a universal approach, it demonstrates the potential of CTCs to expand EC-SPR capabilities in chemical and biomedical sensing.

Figure S6 summarizes the main strategies proposed to overcome technological limitations in EC-SPR sensors. The diagram highlights material innovations (graphene, SAMs, conducting polymers), integration with hybrid techniques (EIS, QCM, ASV), and surface engineering approaches such as molecular imprinting and aptamer-based detection.

Engineering ultrathin films and nanomaterials has been extensively used to optimize sensory detection. Techniques such as LbL assembly and electropolymerization produce highly selective surfaces, with applications in biosensors targeting molecules such as proteins and pesticides.^63,65,74,98,99^

Molecularly imprinted polymers (E-MIPs) have demonstrated high selectivity in the detection of theophylline, achieving detection limits as low as 3.36 μM, thereby reinforcing their potential for biomedical and environmental applications.^19^

Surface functionalization using self-assembled monolayers (SAMs) has proven effective in modulating electrochemical interactions. SAMs containing redox-active groups, such as ferrocene-alkanethiols, enable nanoscale control of sensor response, optimizing the detection of electroactive analytes.^32^

Additionally, copolymer films, such as poly­(pyrrole-*co*-propionic acid) (PPy/PPa), allow for electrostatic adjustments that enhance protein immobilization, thereby increasing the efficiency of immunological biosensors.^32^

Strategies to improve sensor specificity and stability include the use of aptamers for selective detection of antibiotics and biomarkers. The immobilization of aptamers onto gold chips via potential pulses has demonstrated high selectivity for ampicillin, with detection limits of 1 μM, even in complex samples such as river water.^63^

Integrating EC-SPR sensors with advanced optical techniques has been investigated to improve measurement reliability. Fiber-optic sensors with tilted fiber Bragg gratings (TFBGs) have demonstrated high efficacy in detecting heavy metals such as Pb^2+^ at concentrations as low as 10^–10^ M.^81^

Dynamic electrochemical modulation has also been explored to fine-tune sensor response. The generation of silver nanoclusters (Ag^0^) on functionalized SAMs enabled control over surface hydrophobicity and hydrophilicity, enhancing both selectivity and reproducibility.^94^ Furthermore, electropolymerized polypyrrole (PPy) films have been proven effective in detecting anions, such as Cl^–^, establishing a linear relationship between concentration and SPR angle shift, which favors environmental applications.^33^

Simultaneous detection of optical and electrochemical processes has been achieved through the combination of EC-SPR and anodic stripping voltammetry (ASV). This approach has improved the detection of metal ions such as Pb^2+^, Cu^2+^, and Hg^2+^ at subppb levels, eliminating interference and ensuring high precision.^81^

Improving sensor architecture has been key to reducing noise and expanding the electrochemical window. EC-SPR chips based on single-layer graphene (Au/SAM/G) have shown effectiveness in minimizing background signals and enhancing stability under adverse conditions.^98^ Replacing conventional configurations with two-electrode sensors modified with activated carbon has simplified devices, reduced costs, and improved potential stability.^75^

In two-electrode EC-SPR configurations, the term “potential stability” refers to signal reproducibility and baseline steadiness rather than to absolute electrochemical potential control. Although the absence of a reference electrode prevents precise potential calibration, the reduced circuit complexity minimizes parasitic capacitance and electrical noise. Consequently, the optical and electrochemical responses exhibit higher temporal stability and lower drift, which are crucial for the reliable interpretation of EC-SPR signals.

The integration of regenerable sensors offers a promising solution for chemical and biomedical analyses. Dynamic surface regeneration, as demonstrated in the detection of dermatan sulfate (DS) using charge-transfer complexes (CTCs), achieved detection limits of 8 nM, enhancing sensor reliability.^97^

These innovations are driving the advancement of EC-SPR sensors, improving their precision, stability, and applicability across multiple sectors, including clinical diagnostics, environmental monitoring, and analytical chemistry.

## Common Methodologies for Preparation and Characterization of EC-SPR Sensors

The preparation and characterization of EC-SPR sensors rely on the combination of complementary techniques that optimize analyte detection at ultralow concentrations. The integration of high-resolution SPR with anodic stripping voltammetry (ASV) has shown high efficacy in detecting heavy metals at subppb levels. At the same time, the use of miniaturized flow cells and differential systems has improved precision and reduced interference.^77,97,99,100^

Figure S7 summarizes the main methodologies employed for the preparation and characterization of EC-SPR sensors. It illustrates the integration of electrochemical techniques (CV, EIS, ASV), surface characterization methods (AFM, SEM/EDX, XPS), and optical techniques (SPR, UV–vis, Raman), highlighting their complementary roles in optimizing sensor performance.

Chemical vapor deposition (CVD) has been widely used for fabricating highly functional thin films. The Kretschmann configuration maximizes sensitivity in detecting interactions at electrochemical interfaces, allowing for detailed analysis of redox processes.^80^ Optical fiber sensors with tilted fiber Bragg gratings (T1FBGs) enable the real-time integration of optical and electrochemical measurements, demonstrating exceptional sensitivity for Pb^2+^ detection down to 10^–10^ M, making them suitable for remote applications.^100^

Electropolymerization is a key method for synthesizing conductive polymers on electrodes, offering precise control over surface deposition and functionalization. EC-SPR and cyclic voltammetry have been employed to monitor structural changes in ferrocene-alkanethiol-based SAMs, enabling analysis of redox-induced molecular reorientation.^32^ Electropolymerized sensors based on polypyrrole (PPy), polyaniline, and PEDOT/PSS have shown excellent stability and selectivity for biomolecule and heavy metal detection.^16,76,91^

Integration of EC-SPR with electrochemical impedance spectroscopy (EIS), AFM, and XPS has been applied for characterizing functional interfaces, optimizing the detection of biomarkers, pesticides, and antibiotics. Studies have demonstrated that aptamer-modified gold electrodes provide selective detection of ampicillin and glucose, ensuring reproducible and highly sensitive surfaces.^100^

Complementary methods also include FTIR, UV–vis spectroscopy, and AFM for assessing the stability and functionality of polymeric surfaces. The integration of Pb^2+^-dependent DNAzymes with gold nanoparticles (AuNPs) enabled plasmonic and electrochemical signal amplification, resulting in ultralow-level Pb^2+^ detection.^101^

UV–vis spectroscopy and XPS are widely used for characterizing metallic nanocomposites and polymers. XPS has been used to investigate medium-entropy alloy films, revealing the formation of Cr­(OH)_3_, a key factor in the development of corrosion-resistant surfaces.^88^ Raman spectroscopy has enabled the identification of structural defects in graphene and carbon nanotube films, aiding in quality control of EC-SPR materials.^8^

Cyclic voltammetry (CV) has been extensively used for the electrochemical characterization of redox-active materials, allowing for the assessment of the stability and efficiency of conductive polymer films. The formation of solid–electrolyte interface (SEI) layers in lithium-ion batteries has been investigated by combining CV, EC-SPR, and QCM, providing real-time insights into interfacial processes.^80^

Scanning electron microscopy (SEM) and energy-dispersive X-ray spectroscopy (EDX) were used to reveal the uniformity and elemental composition of the nanomaterials used in sensor fabrication. Reduced graphene oxide (ErGO) and metal oxide films demonstrated excellent stability and electrochemical responsiveness.^19^

The integration of optical and electrochemical methods has proven essential for biomedical sensor development, molecular interaction analysis, and environmental monitoring. Advances in multimodal techniques have enabled the development of high-resolution, reproducible sensors, which are critical for the evolution of biosensors and portable analytical devices.

## Results and Discoveries

### Real-Time Monitoring of Electrochemical and Optical Processes

Real-time monitoring of electrochemical and optical processes has been critical to understanding the dynamics of conductive thin films, doping, and molecular interactions. EC-SPR has been widely used to track structural and electronic variations in these materials.

Studies of PEDOT via EC-SPR revealed changes in dielectric constant and optical conductivity, indicating the formation of polaronic bands. Integration with QCM allowed precise determination of film thickness and dielectric variations.^41^ EC-SPR was utilized in the electropolymerization of multilayer films of sexithiophene and carbazole precursors, allowing for the visualization of structural changes and aggregation in real-time. The technique was also applied to the deposition of Hg–Au metal alloys, allowing for detailed tracking of layer formation and alloy composition.^102^

Figure 4 illustrates real-time EC-SPR signal variations during film growth and redox modulation, highlighting typical shifts in resonance angle (Δθ_SPR_) correlated with electrochemical potential (*E*). Examples include PEDOT doping and dedoping, as well as Ag^0^ nanocluster formation.

**Figure 4:**
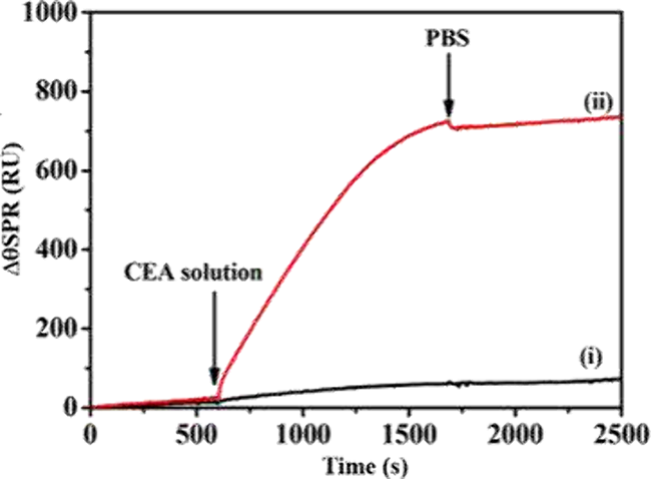
Representative real-time EC-SPR response showing Δθ_SPR_ variations during analyte binding and rinsing steps. The plot illustrates the typical growth-type profile (red curve) and baseline behavior (black curve). Adapted from ref 94. Copyright 2017 American Chemical Society.

### Detection of Biomolecules and Advanced Sensors

EC-SPR has shown remarkable selectivity for adrenaline detection using ultrathin films of poly­(2-aminobenzylamine) (PABA).^74^ The selectivity originates primarily from the specific redox reaction between adrenaline and benzylamine groups within the PABA matrix, which generates distinct electrochemical and plasmonic responses. When compared to interfering species such as uric and ascorbic acids, the EC-SPR signals for adrenaline exhibit apparent differences in both amplitude and phase, confirming that the combined electrochemical–optical interrogation enhances discrimination power beyond either technique alone. Nevertheless, since these interferents differ structurally from adrenaline, further investigation is required to assess how EC-SPR systems perform against compounds with closer molecular similarity, which remains an open question for future optimization. A two-electrode configuration, which eliminates the reference electrode, proved efficient for studying electropolymerization processes and characterizing the deposits, dielectric constants, and redox states of growing films.^15^

EC-SPR has also been applied to study the formation of solid–electrolyte interfaces (SEIs) in lithium-ion batteries, capturing real-time variations in mass and reflection angle, which aids in the optimization of electrolyte additives and electrochemical stability.^80^

Figure S8 schematically summarizes EC-SPR biosensing mechanisms, showing analyte binding (e.g., ascorbic acid, uric acid, ampicillin) and signal transduction in two-electrode configurations. The diagram emphasizes selectivity, interference reduction, and SEI detection.

### Biomolecular Interactions and Redox Processes

Studies on the electrochemical reduction of graphene oxide (GO) demonstrated EC-SPR’s capability to monitor refractive index and resonance angle changes during its conversion to reduced graphene oxide (ErGO), providing valuable insights for biosensor and optoelectronic applications.^9^

The technique was applied to study SAMs of ferrocenylhexanethiol (FcC_6_SH) and ferrocenylundecanethiol (FcC_11_SH), revealing that redox processes induce structural changes and reduce alkyl chain tilt angles, showing EC-SPR’s ability to detect subnanometer conformational reconfigurations.^32^

Moreover, studies on charge-transfer complexes (CTCs) of *o*-tolidine demonstrated greater sensitivity than EQCM for detecting dermatan sulfate (DS), achieving detection limits of 8.^97^

EC-SPR was also used to characterize α-helical leucine-rich peptide monolayers modified with ferrocene, revealing that redox changes significantly alter film thickness due to dipole–dipole interactions, with implications for molecular device development.^35^

Figure 5 illustrates molecular-scale redox-induced conformational changes in SAMs, CTCs, and peptide films. EC-SPR angle shifts and refractive index variations are correlated with molecular tilt, dipole orientation, and film regeneration processes.

**Figure 5:**
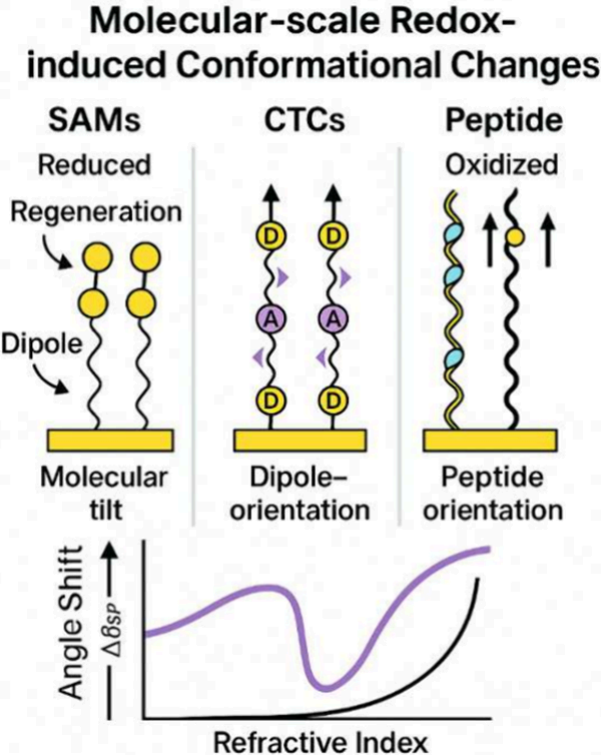
Redox-induced conformational changes in SAMs, CTCs, and peptide films.

### Applications in Electropolymerization and Functional Polymers

EC-SPR has been used to monitor the electropolymerization of polypyrrole (Ppy) thin films, revealing changes in the resonance angle associated with the dielectric constant and film thickness during anion doping and dedoping. The study evaluated the effects of film thickness, charge, and anion size on sensor response.^93^

Redox-dependent interactions between cytochrome *c* (Cyt *c*) and cytochrome *c* oxidase (COX) were investigated, allowing measurement of association/dissociation constants and evaluation of Cyt *c*_red_ and Cyt *c*_ox_ affinities under mitochondrial-membrane-like conditions.^34^

The technique also enabled monitoring of electrochemically mediated atom transfer radical polymerization (eSI-ATRP), demonstrating precise control over polymer initiation and termination by adjusting the substrate potential. The direct correlation between applied potential and polymer growth rate highlights the potential of EC-SPR for advanced polymerization studies.^22^

Similarly, poly­(aminobenzylamine)-based multilayer films prepared via layer-by-layer assembly have demonstrated efficient charge transfer and redox activity for dopamine sensing, reinforcing the potential of aminobenzylamine derivatives for EC-SPR transduction platforms.^103^

Figure 6 illustrates EC-SPR monitoring of electropolymerization, displaying the evolution of the resonance angle as a function of applied potential and film growth. The graph highlights the correlation between polymer thickness, doping level, and optical response.

**Figure 6:**
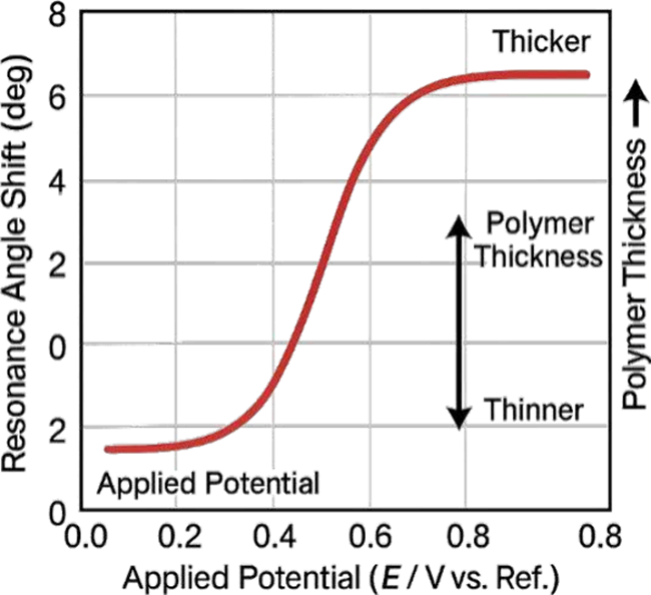
EC-SPR monitoring of electropolymerization dynamics.

Understanding redox processes in EC-SPR is fundamental because the electron-transfer dynamics directly modulate the local refractive index and thus the plasmonic response. Each oxidation or reduction event alters charge density and dipole orientation at the interface, producing measurable optical shifts that encode the chemical state of the surface. This coupling between charge transport and optical permittivity defines the transduction mechanism of EC-SPR sensors. Consequently, characterizing redox behavior enables the design of films and interfaces with optimized sensitivity, faster response times, and greater selectivity toward specific analytes.

## Development and Characterization of Advanced Materials

### Synthesis and Modification of Materials for EC-SPR Sensors

Sriwichai et al.^20^ analyzed ultrathin PEDOT films using EC-SPR combined with QCM to investigate their dielectric constants and electrochromic properties, which are relevant for OLED and electrochromic window applications. The high curvature of gold nanoparticles influences multilayer formation of copper hexacyanoferrate (CuHCF), improving sensor sensitivity.^90^

Modifying electrodes with gold nanoparticles and conductive polymers has been explored to enhance sensor selectivity and stability. Biosensors based on poly­(3-aminobenzoic acid) and poly­(3-carboxypyrrole) enable the simultaneous detection of glucose and immunoglobulin G (IgG), demonstrating high sensitivity for biomedical applications.^21,72^ Similarly, graphene oxide (GO) and PEDOT/PSS-based immunosensors improved antibody immobilization, enhancing biomarker detection specificity.^19^

Figure S9 schematically represents the main material architectures developed for EC-SPR sensors, including polymeric films (PEDOT, PABA, PPy), graphene-based coatings, and gold-nanoparticle interfaces. The diagram highlights how functionalization layers (SAMs, antibodies, aptamers) enhance surface uniformity, stability, and charge-transfer efficiency.

### Surface Functionalization and Structural Characterization

EC-SPR has been used to characterize thiol and carbazole-based SAMs, confirming the formation of highly uniform conjugated polymer networks. The immobilization of anti-IgG and anti-IgM antibodies resulted in highly sensitive and stable surfaces, achieving up to 97% surface coverage.^23^

The development of EC-SPR chips based on Au/SAM/Graphene has led to improved electrochemical stability, reduced background noise, and an extended electrochemical window, thereby preventing issues such as oxidation and delamination commonly seen in conventional chips.^98^

Figure S10 illustrates typical EC-SPR chip structures (Au/SAM/graphene and Au/polymer/nanoparticles) used to improve electrochemical stability and reduce background noise. The figure shows the layer sequence, active binding sites, and electric field distribution near the metal–dielectric interface.

### Advanced Applications and Material Optimization

EC-SPR has also been used to study the selective dissolution of multilayer films of inorganic ions and DNA, enabling controlled DNA release under specific potentials.^33^ Pernites et al.^91^ employed EC-SPR to characterize molecularly imprinted polymers (MIPs), demonstrating high selectivity for theophylline detection.

The technique was applied to detect hydrogen peroxide in gold nanohole devices, allowing for localized measurement of enzymatic reactions for portable diagnostics.^104^ Studies on medium-entropy metal alloys have shown that EC-SPR enables real-time evaluation of anticorrosive film formation.^88^

Bilayer deposition of silver and gold films was optimized to enhance EC-SPR sensitivity, confirming the importance of thickness control in sensor performance.^105^

### Noise Suppression and Sensor Efficiency

Ultrathin PABA films exhibited high selectivity for adrenaline, effectively distinguishing it from interferents like ascorbic and uric acids.^74^ Davis et al.^92^ investigated the modification of ferrocenylalkanethiol SAMs with β-thiocyclodextrin, improving charge-transfer efficiency and reducing electrochemical interference.

EC-SPR was also employed to study charge-transfer kinetics in redox reactions, simplifying kinetic analysis and optimizing sensors for battery and biosensor detection.^106^ Biosensors based on poly­(2-aminobenzylamine) were designed for selective detection of adrenaline, demonstrating the importance of film modification in improving sensor selectivity and accuracy.^74^

EC-SPR remains a vital tool for developing high-resolution sensors, optimizing materials, reducing noise, and enhancing charge-transfer efficiency, thereby ensuring significant advances in chemical and biomedical detection.

## Relationship between Optical and Electrochemical Signals

The integration of optical and electrochemical signals is essential for understanding the behavior of thin films, deposition processes, and redox reactions. The EC-SPR technique enables simultaneous monitoring of these responses and is vital for the development of advanced sensors.

Figure 7 illustrates the correlation between optical and electrochemical signals in EC-SPR. The schematic shows the simultaneous acquisition of reflectivity (Δ*R*) or resonance angle shift (Δθ_SPR_) as a function of applied potential (*E*), highlighting the temporal and kinetic synchronization between electrochemical charge transfer and optical response.

**Figure 7:**
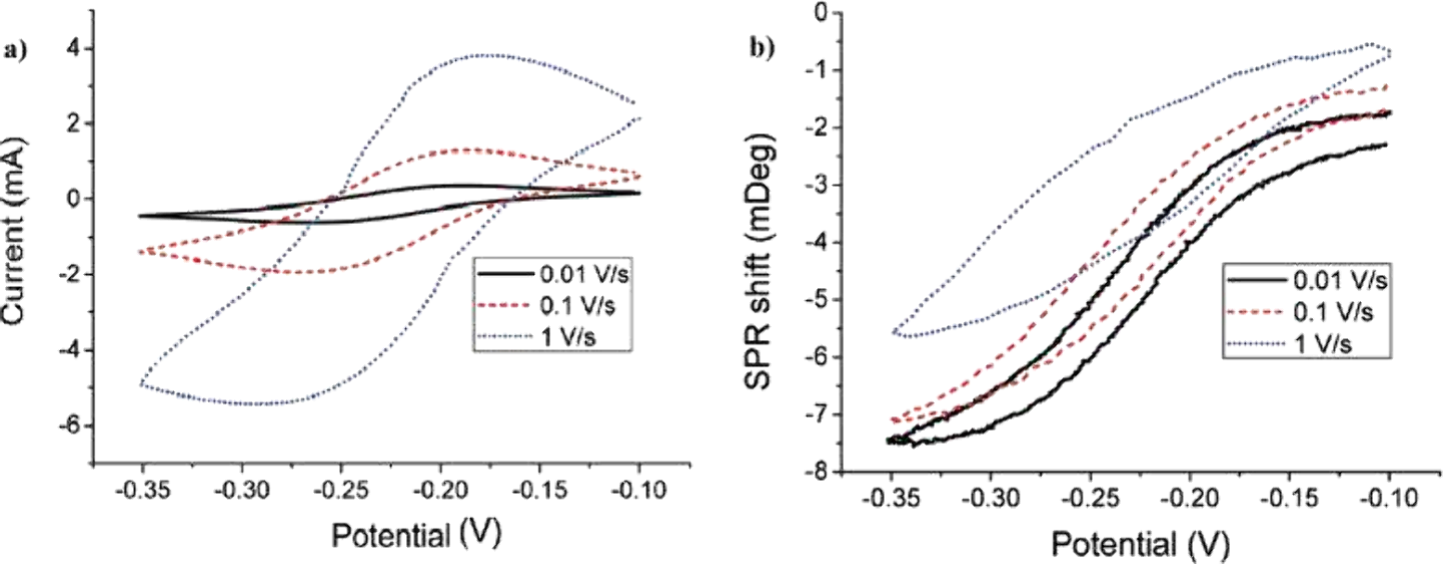
(a) Cyclic voltammetry response recorded at different scan rates. (b) SPR shift as a function of applied potential. Both panels illustrate the kinetic correlation between electrochemical charge-transfer processes and plasmonic modulation. Adapted from ref 18. Copyright 2010 American Chemical Society.

### Origin of the EC-SPR Signal and Electro-optical Coupling Mechanism

The EC-SPR response arises from the modulation of the local dielectric constant (ε) at the metal–electrolyte interface induced by electrochemical charge transfer. When an external potential is applied, electron redistribution in the metal modifies the interfacial electric field and the thickness of the electrical double layer (EDL). These variations alter the refractive index (*n*) within the evanescent field region, typically extending 200–300 nm into the dielectric medium (see Figure 8). As a result, the plasmon resonance condition governed by the Fresnel equations for multilayer systems, shifts in angle (Δθ_SPR_) or reflectivity (Δ*R*). The magnitude of this optical shift is directly correlated with the surface charge density (σ) and the kinetics of electron transfer, allowing for the quantitative monitoring of redox reactions, adsorption, and ion intercalation processes.

**Figure 8:**
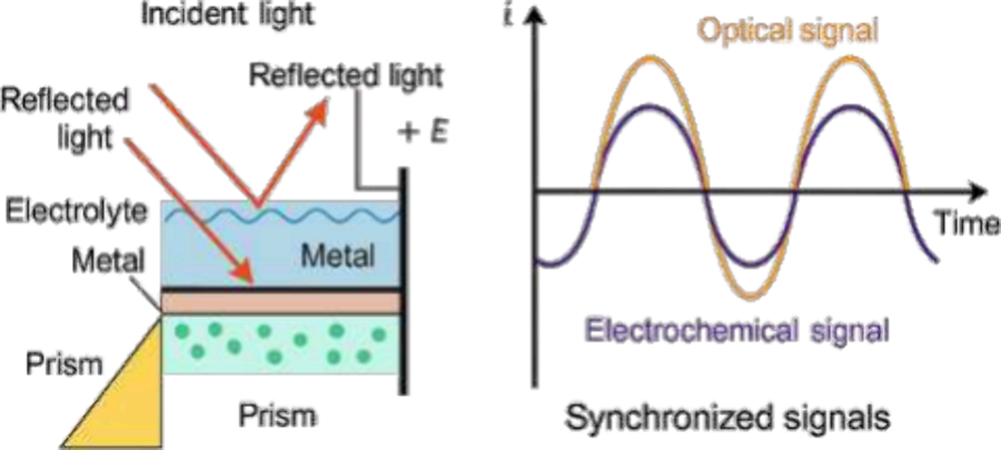
Origin of the EC-SPR signal and electro-optical coupling mechanism.

Therefore, EC-SPR simultaneously captures the electrochemical potential (*E*) and the corresponding optical response, linking charge dynamics to dielectric modulation with subnanometer sensitivity.

### Integration of EC-SPRi for High-Resolution Mapping

Recent developments have extended conventional EC-SPR into imaging-based configurations (EC-SPRi), which employ CCD or CMOS detectors to monitor spatially resolved reflectivity across the electrode surface. EC-SPRi enables the simultaneous acquisition of thousands of individual SPR responses, allowing for the visualization of heterogeneous redox processes, film uniformity, and localized adsorption events. The electrochemical control enhances image contrast by modulating interfacial charge density in real-time, thereby improving the signal-to-noise ratio and spatial resolution compared to optical SPRi alone (see Figure S11). These advances have opened pathways for multiplexed biosensing, microarray analysis, and visualization of nanoscale electrocatalytic activity.

### Monitoring of Deposition and Redox Reactions

EC-SPR has been utilized to investigate the electrochemical deposition of tellurium (Te) on gold substrates, demonstrating that Cd^2+^ ions serve as mediators in the layer-by-layer growth process, leading to more uniform surfaces and controlled deposition rates.^102^

Another study correlated potential sweep voltammetry and EC-SPR measurements to monitor the redox reactions of ruthenium­(III) hexammine chloride, demonstrating a direct relationship between the signals, thus allowing for kinetic reaction analysis.^18^

The formation of copolymerized films of polysiloxane/polythiophene was monitored using EC-SPR and electrochemical impedance spectroscopy (EIS), highlighting structural changes during deposition.^93^ Similarly, immunosensors based on polypyrrole with propionic acid demonstrated that the combination of EC-SPR and EIS allows detailed monitoring from polymer formation to biomolecular detection.^33^

### Applications in Electrochemical Devices and Sensors

EC-SPR has been employed in the detection of uric acid using conductive films composed of single-walled carbon nanotubes (SWNTs), correlating structural properties with plasmonic signals.^30^

In lithium-ion battery studies, the combination of EC-SPR and quartz crystal microbalance (QCM) enabled the analysis of solid–electrolyte interface (SEI) formation by monitoring mass variation, reflection angle, and intensity during redox processes.^80^

Detection of amphetamine-type stimulants using EC-SPR has demonstrated the technique’s sensitivity in capturing refractive index changes, highlighting its importance in selective sensor development.^83^ Additionally, studies on van der Waals heterostructures (vdW-HS) have shown that graphene on hexagonal boron nitride enhances the sensitivity of redox detection.^78^

### Impact on Sensor Sensitivity and Selectivity

The combination of EC-SPR and biosensors has shown significant gains in the simultaneous detection of glucose and human IgG, correlating optical and electrochemical responses to enhance sensor accuracy.^20^

Sensors based on graphene oxide and PEDOT/PSS have expanded selectivity in biomolecule detection.^19^ Similarly, EC-SPR immunosensors have been applied to influenza virus detection, using electrochemical potentials to amplify the optical signal, making the technique crucial for biomedical diagnostics.^77^

### Differences between Optical and Electrochemical Signals

Although many reactions exhibit a direct correlation between optical and electrochemical signals, others present time or amplitude discrepancies due to differences in interfacial dynamics, particularly in diffusion- or adsorption-dominated processes.^75,104^

In EC-SPR systems, the applied potential modulates the surface charge density of the metal film, altering the local dielectric constant and, consequently, the resonance condition. For charge-transfer-controlled reactions, such as ferrocene oxidation or PEDOT doping, the reflectivity (Δ*R*) and current (*I*) evolve synchronously, revealing direct coupling between electron transfer and refractive index modulation. Conversely, in protein adsorption or ion intercalation processes, the electrochemical current may stabilize before the optical signal, as molecular rearrangements and solvent relaxation occur more slowly than electron flow. This leads to phase shifts or amplitude mismatches between Δ*R* and *I*, which provide additional information about kinetic or structural mechanisms at the interface.

Studies involving DNA probes immobilized on electrodes showed that molecular orientation influences both plasmonic and electrochemical responses, reinforcing the importance of detailed material characterization in EC-SPR sensor development.^108^

Figure S12 compares correlated and noncorrelated signal responses in EC-SPR systems. While direct redox processes yield synchronized variations in both optical and electrochemical signals, diffusion- or adsorption-dominated mechanisms show phase delays or amplitude mismatches, requiring careful data interpretation.

### Nonredox and Imaging Approaches in EC-SPR

While most EC-SPR studies focus on electrochemically active probes, several investigations have demonstrated that valuable information can be obtained even in the absence of redox-active species. In such cases, the applied potential modulates interfacial charge distribution, affecting the orientation, hydration, or adsorption of neutral or dielectric layers. These systems enable quantitative assessment of protein adsorption, lipid membrane formation, and thin-film dielectric properties without requiring electron transfer reactions.^32,33^

This nonredox EC-SPR approach provides a unique advantage over conventional electrochemistry and SPR alone, as it combines the electrical tunability of the interface with high optical sensitivity to nanometric structural variations.

In parallel, coupling EC-SPR with surface plasmon resonance imaging (EC-SPRi) has expanded the technique toward spatially resolved analysis. EC-SPRi enables the simultaneous mapping of optical and electrochemical activity across the electrode surface, allowing for the visualization of heterogeneous adsorption, film growth, or catalytic reactions in real-time.^18,78^

This coupling represents a significant step forward in the development of multiplexed sensors and microarray-based biosensing platforms, bridging electrochemical control and optical imaging with micrometer-scale precision.

### Practical Applications

EC-SPR has been used to monitor the electrochemical polymerization of ultrathin layers of sexithiophene and polycarbazole, allowing the tracking of structural changes during film growth.^91^

In environmental applications, EC-SPR has proven effective in detecting metal ions and monitoring adsorption/desorption processes on metallic surfaces, providing essential information for the development of heavy metal sensors.^90^

The technique continues to evolve, enabling significant advances in biomedical sensors, electrochemical devices, and environmental analysis, establishing itself as a key tool for the simultaneous monitoring of chemical reactions and structural properties.

## Instrumentation and Cell Configuration in EC-SPR Systems

The electrochemical surface plasmon resonance (EC-SPR) configuration integrates a classical electrochemical cell with the optical Kretschmann setup, where a prism couples light into a thin metallic film, typically gold, deposited on a glass substrate. This gold layer functions simultaneously as both the plasmonic transducer and the working electrode. The electrochemical cell is designed to ensure optical transparency, chemical compatibility, and mechanical stability, generally employing Teflon or PMMA housings, O-ring seals, and fluidic channels to control electrolyte flow and prevent leakage.

### Electrode Materials and Geometry

In most configurations, a 40–50 nm gold film is sputtered onto a glass or quartz slide to ensure optimal plasmonic resonance and electrical conductivity. Platinum or carbon electrodes are commonly used as counter electrodes, while Ag/AgCl or saturated calomel electrodes serve as stable references. For compact or portable systems, pseudoreference electrodes made of gold or platinum are often employed to simplify integration. Electrode positioning and geometry strongly influence local electric fields and optical coupling, directly affecting sensitivity, noise level, and reproducibility.

### Two- versus Three-Electrode Configurations

Traditional EC-SPR systems employ a three-electrode configuration to achieve precise potential control and minimize ohmic drop. However, simplified and miniaturized platforms increasingly utilize a two-electrode configuration, which eliminates the need for a reference electrode and reduces circuit complexity and electronic noise.

The effectiveness of the two-electrode configuration was demonstrated by monitoring the in situ electropolymerization of polyaniline films using EC-SPR, showing that the same gold substrate can simultaneously function as the working and pseudoreference electrode while preserving high optical sensitivity and electrochemical stability.^15^ This configuration simplifies the instrumentation and improves reproducibility, making it particularly suitable for portable EC-SPR systems and microfluidic integration.

Further studies have extended the two-electrode concept to biosensing and thin-film analysis, where reduced electrode count improves noise performance and compactness without significant loss of accuracy.^15^

### Commercial and Custom EC-SPR Systems

Several commercial instruments currently integrate synchronized electrochemical and optical acquisition, such as the BioNavis SPR Navi Multi-Parameter, Reichert SPR-E2, and Horiba EC-SPR modules. These systems allow simultaneous impedance or cyclic voltammetry measurements during plasmonic monitoring, providing robust and reproducible experimental conditions. In research laboratories, custom-built setups remain widely used, particularly for coupling EC-SPR with complementary techniques such as electrochemical impedance spectroscopy (EIS), quartz crystal microbalance (QCM), or Raman spectroscopy. The modular design of EC-SPR setups facilitates adaptation for studies in catalysis, corrosion, biosensing, and thin-film characterization.

The combined discussion of cell architecture, electrode design, and instrumentation highlights how the experimental configuration governs EC-SPR sensitivity, reproducibility, and scalability. Understanding these parameters is essential for optimizing system stability and enabling future integration with imaging modules (EC-SPRi) and microfluidic platforms for high-throughput applications.

## Surface Functionalization for Biosensing and Heavy Metal Detection

Surface functionalization is crucial in EC-SPR sensor development, as it enhances selectivity and sensitivity for the detection of biomolecules and heavy metals. Biomolecule immobilization and the integration of optical and electrochemical techniques have led to significant advancements in environmental monitoring and biomedical diagnostics.^65,76^

Figure S13 schematically represents the biomolecule immobilization mechanisms in EC-SPR biosensors, illustrating SAM-based and polymer-functionalized surfaces for the attachment of antibodies, antigens, and aptamers. The figure illustrates signal generation from redox-induced refractive-index changes, highlighting applications in glucose, IgG, and antibiotic detection.

### Biosensors and Biomolecule Detection

EC-SPR has been applied to the simultaneous detection of glucose and human IgG through the immobilization of biomolecules on carboxylated conductive polymer films, demonstrating high selectivity.^20^ Furthermore, PEDOT/PSS and graphene oxide-based sensors have been developed to enhance antigen capture efficiency, particularly for influenza virus detection and clinical biomarker monitoring.^77^

The use of EC-SPR aptasensors for antibiotic detection, such as ampicillin, has achieved detection limits as low as 1 μmol L^–1^, confirming their suitability for environmental and clinical applications.^63,76,94^

### Heavy Metal Detection

Integrating SPR with anodic stripping voltammetry (ASV) has enabled the simultaneous detection of Pb^2+^, Cu^2+^, and Hg^2+^ with high selectivity and sensitivity in the range of 10^–10^ M to 10^–5^ M.^90,100^ The use of gold-coated fiber-optic sensors improved measurement precision, enabling applications in remote and hard-to-access environments.^76^

Figure S14 illustrates the integration of EC-SPR with anodic stripping voltammetry (ASV) for simultaneous detection of heavy metals (Pb^2+^, Cu^2+^, Hg^2+^). The schematic shows electrodeposition, stripping peaks, and the corresponding SPR angle shifts (Δθ_SPR_), highlighting the correlation between electrochemical and optical responses.

### Film Deposition and Stability

Monitoring the deposition of polymeric films, such as PEDOT and polypyrrole, has been optimized via EC-SPR, allowing for real-time control of thickness and dielectric properties, which are crucial for optoelectronic device applications.^93^ Studies have revealed that reduced graphene oxide (ErGO) and PEDOT/PSS films exhibit high stability and reproducibility, which are essential for long-term sensors.^9,20^

### Applicability in Advanced Sensors

EC-SPR has been utilized in the characterization of DNA sensors, enabling the differentiation between specific hybridization and nonspecific contaminant adsorption, a crucial factor for precise molecular diagnostics.^108^ GO/PEDOT/PSS-based sensors have demonstrated high sensitivity for human IgG detection, making them promising for clinical applications.^19^ Combining EC-SPR with electrochemical and optical techniques has optimized sensor stability, selectivity, and sensitivity, expanding applications in biomedical diagnostics, environmental analysis, and optoelectronic device development.

## Enhancing Sensors with Advanced Data Processing Techniques

### Electrode and Film Modifications for Sensors

Electrode functionalization with gold nanoparticles and mixed ferrocene alkanethiol/β-cyclodextrin systems has demonstrated stability and efficient electron transfer, which is essential for high-precision sensors.^32^ Electrochemical copolymerization of terthiophene and carbazole films enhanced electrochromic and mechanical properties, optimizing optoelectronic devices.^91^

The layer-by-layer (LbL) technique was applied to create multilayer films of gold nanoparticles and DNA, ensuring stability and uniformity for sensors and controlled release systems.^85^ Polypyrrole sensors modified with *N*-alkylamine and glucose oxidase (GOx) demonstrated high sensitivity for glucose detection (10 mM), with notable selectivity for bioelectronic analysis.^21,33^

### Data Processing for EC-SPR Sensors

Advanced techniques, such as the Karhunen–Loève transformation (KL), which is similar to principal component analysis (PCA), have been used to process SPR curves without manual adjustment. This approach enhanced the detection of small molecules, such as H_2_O_2_ and glutamate, thereby optimizing sensor precision.^84^ Mathematical models have correlated electrochemical and EC-SPR signals, validating theoretical predictions regarding sensor behavior under various experimental conditions.^18^

Figure S15 schematically represents the data processing workflow in EC-SPR analysis. It demonstrates the simultaneous acquisition of electrochemical and optical signals, preprocessing (including noise filtering and baseline correction), feature extraction (Δθ_SPR_ and current peaks), and dimensionality reduction through KL/PCA transformation for pattern recognition and multivariate correlation.

### Correlation between Optical and Electrochemical Signals

In EC-SPR, the correlation between plasmonic and electrochemical responses arises from the coupling between interfacial charge density and local dielectric permittivity. During redox processes, electron transfer at the working electrode alters the surface charge distribution and induces ion rearrangement within the electrical double layer. These changes modify the refractive index near the metal–dielectric interface, shifting the plasmon resonance angle (Δθ_SPR_) or reflectivity (Δ*R*). Simultaneously, the electrochemical signal (current or potential) reflects the kinetics of electron exchange. Therefore, time-synchronized acquisition of both signals enables direct mapping of optical variations to faradaic processes, allowing the determination of charge-transfer rates, diffusion regimes, and adsorption/desorption mechanisms with nanometric precision. This quantitative correlation transforms EC-SPR from a descriptive optical probe into a dual-domain analytical platform capable of resolving dynamic interfacial processes in real time.

The integration of EC-SPR and cyclic voltammetry (CV) enabled the study of the electrocatalytic oxidation of ascorbic acid in PEDOT films, demonstrating that polymer doping can be modulated by applying positive and negative potentials.^19^

Monitoring the orientation of 11-ferrocenylundecanethiol (FcC_11_SH) monolayers revealed how redox changes influence molecular reorganization and water incorporation, affecting the dielectric constant of the film.^32^

EC-SPR was also applied to study solid–electrolyte interface (SEI) formation in lithium-ion batteries, correlating optical and electrochemical signals to monitor ion dissociation rates and passivation layer formation.^79,80^

### Advances in Biosensors and Metal Detection Sensors

EC-SPR sensors based on poly­(2-aminobenzylamine) (P2ABA) enabled the accurate detection of adrenaline, with a limit of detection of 10 pM, effectively distinguishing it from interferents such as uric acid.^74^ Bioinert surfaces employing oligoethylene glycol (OEG)-functionalized carbazole dendrons demonstrated resistance to protein adsorption, critical for selective biomedical sensors.^109^

### Interference Reduction and Computational Validation

Using SAMs of ferrocene alkenethiol and β-cyclodextrin reduced interference, allowing for precise discrimination between different redox states.^110^ The computational validation of EC-SPR sensors was enhanced by the KL method, enabling the precise identification of minor refractive index variations and improving the reliability of environmental and biomedical analyses.^84^

## Effectiveness of the Two-Electrode Configuration Compared to the Three-Electrode Setup

### Comparison Between Two- and Three-Electrode Configurations

The choice between two- and three-electrode configurations is a critical design factor in EC-SPR systems, as it directly influences measurement stability, reproducibility, and optical–electrochemical coupling.

Traditional three-electrode cells offer precise potential control; however, their geometry often leads to increased optical path distortion and signal noise. In contrast, simplified two-electrode configurations minimize instrumental complexity, enhance optical alignment, and facilitate integration into miniaturized or portable sensor platforms. Understanding how these configurations affect plasmonic and faradaic responses is crucial for optimizing EC-SPR device architecture, reducing background interference, and enabling real-time, field-deployable applications.

Studies have compared the efficiency of two- and three-electrode configurations in electrochemical sensors and optoelectronic devices. The two-electrode setup proved effective for the copolymerization of thiol and polysiloxane monomers conjugated with polythiophene, enabling precise control of deposition and dielectric properties.^91^

Another study employed polypyrrole films with propionic acid (PPA) in a two-electrode system for monitoring immunosensors using EC-SPR. Results showed that this configuration was sufficient to track antigen–antibody reactions while maintaining sensitivity and precision comparable to the traditional three-electrode system.^32^

Figure 9 schematically compares the conventional three-electrode configuration (working, reference, and counter electrodes) with the simplified two-electrode setup commonly used in EC-SPR. The figure highlights the elimination of the reference electrode, the current flow path, and how the potential control remains stable through electrode material optimization (e.g., activated carbon).

**Figure 9:**
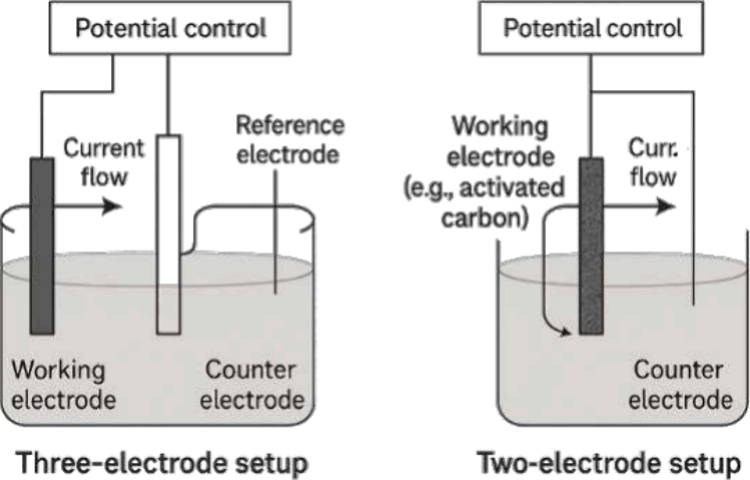
Comparison between three-electrode and two-electrode EC-SPR configurations.

### Validation of the Two-Electrode Configuration

The two-electrode configuration was validated for the polymerization of polyaniline (PAn), using an activated carbon electrode as the counter electrode, eliminating the need for a reference electrode. Results confirmed that this setup maintained a stable potential, allowing for detailed monitoring of dielectric constant changes and polymer film thickness.^10^

### Differences in Resolution and Sensitivity

The two-electrode configuration demonstrated advantages in experimental simplification and interference reduction without compromising resolution. In a study on uric acid detection using composite films of poly­(2-aminobenzylamine) (P2ABA) and single-walled carbon nanotubes (SWNTs), the sensors exhibited high selectivity even in the presence of interferents such as ascorbic acid.^16^

Moreover, immunosensors based on poly­(pyrrole-3-carboxylic acid) (PP3C) for human IgG detection showed comparable responses to those of three-electrode systems, simplifying instrumentation and improving detection efficiency.^20^

### Practical Applications and Robustness

The two-electrode configuration has been extensively validated for doping conductive films and real-time monitoring of redox reactions. A study on the electropolymerization of PAn via EC-SPR showed that this configuration simplified the experiment while enabling the tracking of polymer redox transitions, maintaining data integrity.^75^

Another study used PP3C and carbon nanotube-based sensors to monitor antigen–antibody interactions. The configuration provided accurate measurements even at extremely low concentrations, proving its feasibility for biomedical applications.^33^

## Weaknesses and Gaps in EC-SPR Research

### Limitations in Material and Method Selection

Studies focused on specific materials, such as self-assembled monolayers (SAMs) of 11-ferrocenylundecanethiol (FcC_11_SH), limit the generalizability of results. Additionally, some studies analyzed only bilayer silver/gold films without exploring other metallic combinations that could expand the technique’s applicability.^32,105^

The lack of comparison with other analytical techniques, such as electrochemical impedance spectroscopy (EIS) or differential pulse voltammetry, weakens the assessment of EC-SPR’s effectiveness.^30^ Furthermore, many studies have not evaluated the impact of variations in pH, temperature, or analyte concentration, which compromises sensor reproducibility and real-world applicability.^19,91^

Figure S16 summarizes the main weaknesses and gaps identified in EC-SPR research. The conceptual map groups limitations into four major categories: material constraints, methodological challenges, lack of quantitative validation and industrial and economic barriers. Each category highlights representative issues and their interdependencies, illustrating how technical and economic limitations jointly affect reproducibility and scalability.

Experimental complexity limits reproducibility. Studies using gold nanoparticles and CuHCF multilayers require strict control of variables, making replication difficult.^92^ Likewise, graphene-based sensors often require advanced printing and characterization techniques that are not accessible to many laboratories.^98^

Reliance on sophisticated equipment, such as EC-SPR and EC-AFM, restricts the dissemination of results. Precise control of electrochemical parameters is also required, making electropolymerization and redox processes highly sensitive to minor methodological variations.^14,18,31^

### Lack of Quantitative Data and Testing in Real Conditions

Many studies lack detailed quantitative data, such as detection efficiency and quantification limits, preventing objective comparisons with other methodologies.^19,76,77^ Furthermore, most tests were conducted only in standard solutions, neglecting real samples such as blood or contaminated water, which limits the sensor’s applicability in practical scenarios^85,92,98^

### Absence of Economic Considerations and Industrial Viability

Studies often overlook fabrication costs and scalability. Electrode modification with specific polymers may increase production costs, while electrochemical deposition processes require complex infrastructure, hindering large-scale adoption.^30,31^

The industrial application of nanostructured films still faces challenges, such as maintaining uniformity and quality in mass production. Electrode modification methods and thin-film chemical deposition need scalable solutions to become commercially viable.^20,87,98^

### Hybrid Sensing Metrological Gap

Although EC-SPR has matured into a widely used hybrid platform for probing potential-controlled interfacial processes and for augmenting SPR readouts with electrochemical control, the published record remains comparatively sparse in explicitly metrological treatments—notably in studies that formalize the measurand, establish metrological traceability (optical and electrochemical), and report complete uncertainty budgets for the coupled measurement. This imbalance is visible in the way the field is commonly framed: EC-SPR is predominantly reviewed and reported in terms of configuration, operating conditions, mechanistic interpretation, and application demonstrations, rather than in terms of calibration hierarchies and interlaboratory comparability. Addressing this gap motivates three domain-specific metrological research directions. The first is biosensing EC-SPR, which involves the development of traceable optical-channel calibration protocols that map the instrument response (angle/wavelength/intensity units) to a clearly defined surface quantity (e.g., surface mass density or surface coverage) under explicitly stated optical models and with propagated uncertainties. The second is battery/interphase EC-SPR, which involves the creation of traceable operando methodologies that separate potential-driven optical baseline shifts (double-layer charging, electrolyte refractive-index changes, roughness evolution) from true interphase/film growth, supported by reference-electrode control, temperature/RI control, and model-based uncertainty propagation. The third one is general electroanalytical EC-SPR, which involves establishing cross-modal validation workflows that reconcile charge-based quantification (Faradaic charge → amount via Faraday’s law, with quantified Faradaic efficiency) with SPR-derived interfacial optical changes to yield a single, explicitly defined measurand with a defensible uncertainty budget suitable for interlaboratory comparison.

## Advances and Future Needs

The evolution of EC-SPR technology can be represented as a logical sequence of technical improvements, challenges, and proposed solutions. The following flowcharts synthesize this trajectory, highlighting key milestones and future perspectives.

### Conceptual Integration of EC and SPR

Figure S17 illustrates the theoretical and technological foundation that enabled the integration of electrochemistry (EC) and surface plasmon resonance (SPR). It begins with recognizing the limitations of each technique in isolation: EC provides precise data on redox processes, while SPR offers highly sensitive optical detection but lacks electrochemical selectivity. Merging these approaches allowed simultaneous monitoring of optical and electrochemical signals, resulting in a new class of hybrid sensors with high specificity, sensitivity, and applicability in complex systems.

Technological advances in EC-SPR sensors have been driven by improvements in materials and the integration of analytical methods. The combined use of electrochemical and optical techniques has enabled the detection of more precise surface events, especially in complex systems with multiple analytes. Nanomaterials, such as graphene and metallic nanoparticles, have significantly enhanced sensor sensitivity by offering a greater surface area, superior electronic properties, and improved interaction with biomolecules. Simultaneously, hybrid composites formed by conductive polymers and nanomaterials have enhanced sensor system stability and selectivity while facilitating functionalization.

These advances have broadened the applications of EC-SPR sensors in various sectors, including environmental monitoring (e.g., water quality and detection of organic pollutants), healthcare (e.g., biomarker identification for emerging diseases), and indoor air quality analysis (e.g., VOCs and hazardous gases). Other applications include cosmetic sensors for rapid detection of allergic reactions and agricultural systems for assessing nutrient and soil conditions in the field. These innovations underscore the versatility and potential of EC-SPR technology as an adaptable and high-performance sensing platform for real-world monitoring and diagnostics (see Figure S18).

### Strategic Applications and Enhanced Sensor Performance

Improvements in the sensitivity and specificity of EC-SPR sensors have expanded their applications in critical areas, such as healthcare, environmental protection, and food safety. One of the most significant achievements is the rapid detection of antibiotic-resistant pathogens, which enables more effective clinical responses and prevents the spread of multidrug-resistant strains. Immunosensors used to monitor cellular therapies represent another breakthrough, enabling real-time evaluation of the safety and efficacy of advanced treatments such as immunotherapy and gene therapy. Additionally, EC-SPR technology has enabled the detection of rare and genetic diseases with high accuracy, contributing to early and personalized diagnosis.

Performance enhancements also translate to environmental and food applications. Real-time detection of microbial contamination and foodborne chemical residues (e.g., allergens, preservatives) ensures improved quality control and consumer safety. Identifying microplastics and complex organic pollutants in water bodies and soils represents a crucial advancement for environmental monitoring. Sensors with high specificity are also being used to monitor the physiological status of transplanted organs before and after procedures, providing critical support for immediate clinical interventions. Ultimately, integration with personalized nanomedicine enables the development of sensors tailored to individual biological profiles, thereby enhancing the customization of therapies. These applications demonstrate how improving sensitivity and specificity significantly expands the role of EC-SPR sensors in various strategic fields of science and technology (see Figure S19).

### Advances in Structural and Electrochemical Characterization

Advances in EC-SPR sensors have significantly extended the frontiers of materials science and bioengineering. Combining EC-SPR with complementary techniques, such as electrochemical impedance spectroscopy (EIS) and atomic force microscopy (AFM), has enabled the construction of hybrid sensors that provide simultaneous information on optical, electrical, and morphological processes. This integration enhances sensitivity and analytical resolution, particularly for transformations that occur at the molecular or nanoscale level.

Real-time monitoring of complex redox reactions has been crucial for developing energy storage devices, such as batteries and supercapacitors, enabling direct observation of electrochemical and structural changes in electrodes. Additionally, integration with vibrational spectroscopy (Raman and IR) has enabled the chemical and structural analysis of reaction mechanisms. Practical applications include the following:

- sensors capable of detecting multiple biomarkers simultaneously;
- evaluation of surface degradation in corrosive environments;
- sensors for catalytic efficiency assessment in energy conversion;
- monitoring interactions between nanoparticles, biomolecules, and cells.

These applications confirm the potential of advanced structural and electrochemical characterization in driving innovation across catalysis, biomaterials, nanomedicine, and surface science (see Figure S20).

### Technological Challenges in Adverse Conditions

Figure S21 presents the significant technological challenges in developing EC-SPR sensors for extreme environments. A key challenge lies in utilizing advanced materials to enhance signal quality and minimize interference in complex electrochemical systems, an essential requirement for accurate data acquisition in high-noise or variable environments. Real-time monitoring during redox reactions is another barrier, requiring highly responsive and chemically stable sensors. Increasing sensor robustness is also necessary for operation under extreme temperatures, humidity, salinity, or chemical interference. Other challenges include the following:

- extending sensor lifespan;
- developing energy-autonomous sensors (e.g., renewable energy sources);
- ensuring reliable operation in extreme environments such as outer space or deep-sea locations.

Each context demands specific innovations in materials, sensor architecture, and calibration protocols, significantly broadening the scope of EC-SPR research beyond laboratory environments.

### Future Research Trends

Figure 10 highlights the key future trends in EC-SPR sensor development across emerging contexts in science, medicine, agriculture, and industry. One of the most promising directions is sensor miniaturization and portability, aiming to create accessible, real-time devices usable in remote environments. This includes integration with wearable technologies for continuous health monitoring, such as tracking circulating biomarkers outside clinical settings.

**Figure 10:**
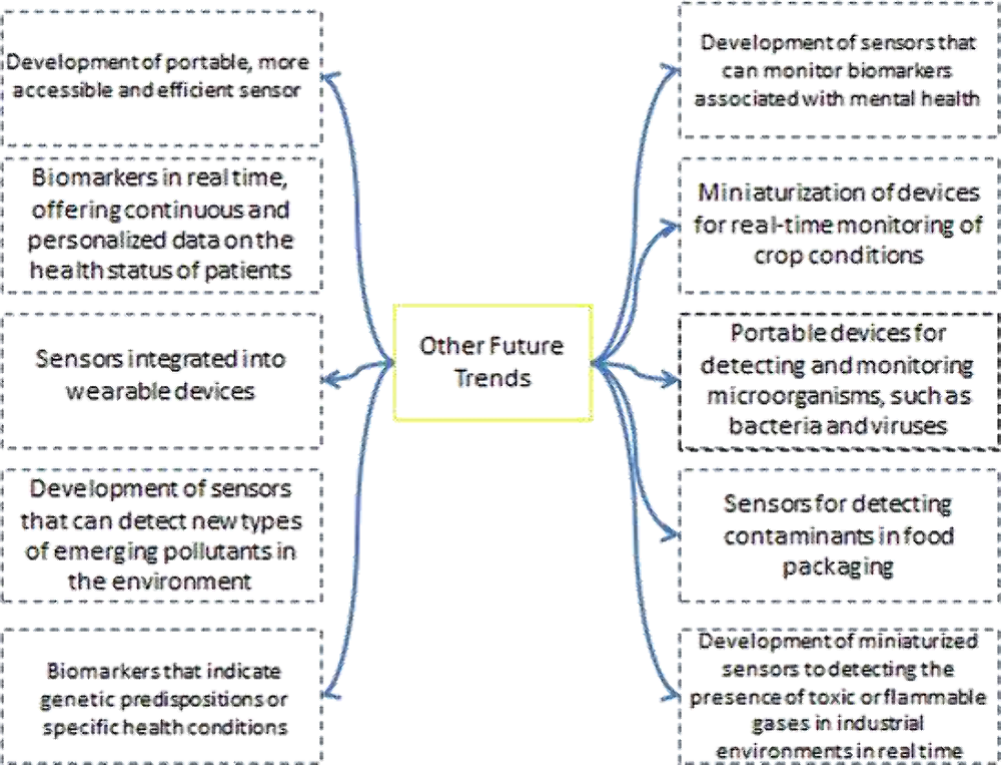
Future trends in EC-SPR sensor development.

Real-time monitoring of biomarkers related to mental health, genetic conditions, and chronic diseases is emerging as a strategic application, enabling early intervention and personalized therapies. In environmental monitoring, new sensors are expected to detect emerging pollutants, including microcontaminants and substances that evade detection by conventional technologies.

Precision agriculture may benefit from miniaturized sensors that directly monitor field conditions. Other trends include sensors for the following applications:

- detecting contaminants in food packaging;
- real-time pathogen monitoring (e.g., viruses and bacteria), supporting epidemiological prevention;
- detecting toxic or flammable gases in industrial settings with high selectivity and immediate response.

These perspectives underscore the adaptability and relevance of EC-SPR sensors in addressing global, interdisciplinary challenges.

## Conclusion

The electrochemical surface plasmon resonance (EC-SPR) technique has been consolidated as a powerful tool for characterizing surfaces, interfaces, and electrochemical processes with high sensitivity and resolution. By integrating the principles of optical spectroscopy with classical electrochemistry, EC-SPR enables real-time monitoring of complex phenomena such as molecular interactions, redox reactions, and structural modifications in functional films. This versatility makes the technique particularly promising in biosensing, catalysis, environmental monitoring, clinical diagnostics, and the development of advanced materials.

Throughout this review, the theoretical foundations, methodological advances, and most relevant applications of EC-SPR have been discussed, with emphasis on the use of hybrid materials, integration with complementary techniques (such as cyclic voltammetry, electrochemical impedance spectroscopy, vibrational spectroscopies, and atomic force microscopy), and strategies for biomolecule immobilization on metallic surfaces. These innovations have significantly improved the sensitivity, selectivity, and robustness of sensors developed with this approach.

However, several challenges remain, including the need for greater sensor reproducibility, the influence of interferents in real matrices, adaptation to extreme operating conditions, and limitations in device lifespan. Advances in materials science, microfabrication, and automation are crucial for overcoming these barriers, as is the development of autonomous and more accessible sensors for field applications.

Future trends indicate an increase in miniaturization, the integration of mobile and wearable devices, and the incorporation of artificial intelligence algorithms for automatic signal interpretation. There is also a growing focus on emerging applications, such as detecting mental health biomarkers, food contaminants, industrial toxic gases, and complex environmental pollutants.

EC-SPR represents a strategic intersection between fundamental science and technological innovation, bringing together tools that, once properly refined, can meet increasingly sophisticated and urgent demands in the coming decades. The consolidation of its large-scale application will depend on interdisciplinary collaboration among researchers, engineers, and industry, in pursuit of efficient, sustainable, and user-integrated solutions.

EC-SPR stands as a cornerstone in the next generation of electrochemical and optical sensing technologies. Its continuous evolution, driven by nanomaterials, AI-assisted data interpretation, and portable architectures, is expected to transform real-time chemical and biological monitoring into a ubiquitous, intelligent, and sustainable process.

## Supplementary Material



## Notes

Schematic representations of EC-SPR sensor architectures; additional illustrations of electrochemical cell configurations and SPR excitation geometries; extended conceptual diagrams of EC-SPR coupling mechanisms; comparative signal-response representations; supplementary figures detailing biomolecule immobilization strategies; integration of EC-SPR with anodic stripping voltammetry for heavy-metal detection; data-processing workflow illustrations; conceptual maps of limitations, research gaps, and technological challenges (PDF)
