# Supplementary material for: Development and Applications of Electrochemical Surface Plasmon Resonance (EC-SPR)-Based Sensors: A Review
Source: Anal Chem. 2026 Mar 9;98(11):7937–58. doi: 10.1021/acs.analchem.5c07065 (PMC13019431; doi:10.1021/acs.analchem.5c07065)
Supplement: Supplementary file 1 [file ac5c07065_si_001.pdf]

Development and Applications of Electrochemical Surface Plasmon Resonance (EC-SPR)-Based Sensors: A Review

Jomar Sales Vasconcelos,[*,†] Nazaré do Socorro Lemos Silva Vasconcelos,[‡] Cícero Wellington Brito Bezerra,[¶] and Antonio Marcus Nogueira Lima[§]

†*Department of Electrical and Electronic Engineering, Federal Institute of Maranhão, Av. Getúlio Vargas, 4, Monte Castelo, 65030-005, Brazil*

‡*Department of Chemistry, Federal Institute of Maranhão, Av. Getúlio Vargas, 4, Monte Castelo, 65030-005, Brazil*

¶*Department of Chemistry, Federal University of Maranhão (UFMA), Avenida dos Portugueses, 1966, São Luís, MA, 65080-805, Brazil*

§*Department of Electrical Engineering, Federal University of Campina Grande (UFCG), Rua Aprígio Veloso, 882, Universitário, Campina Grande, PB, 58429-900, Brazil*

E-mail: *jomar@ifma.edu.br

Supporting Information

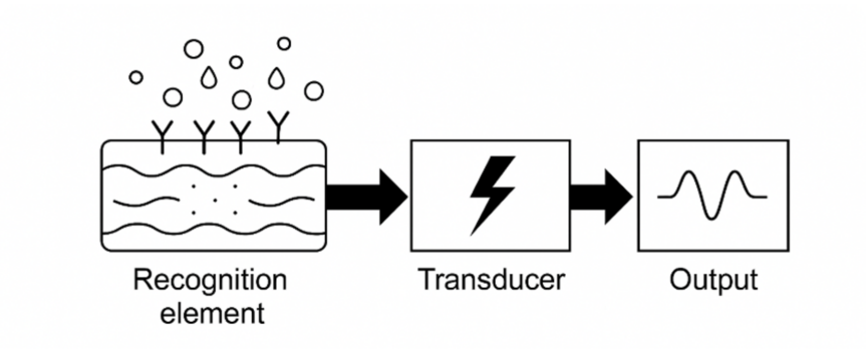

Figure S1: General architecture of a sensor.

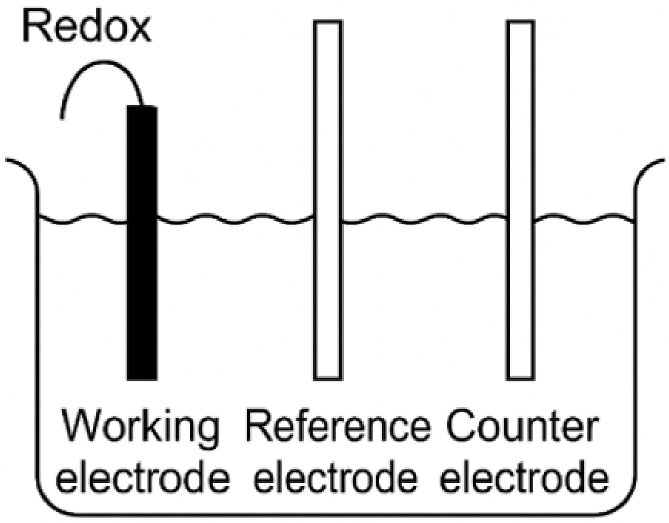

Figure S2: Schematic representation of a conventional three-electrode electrochemical cell.

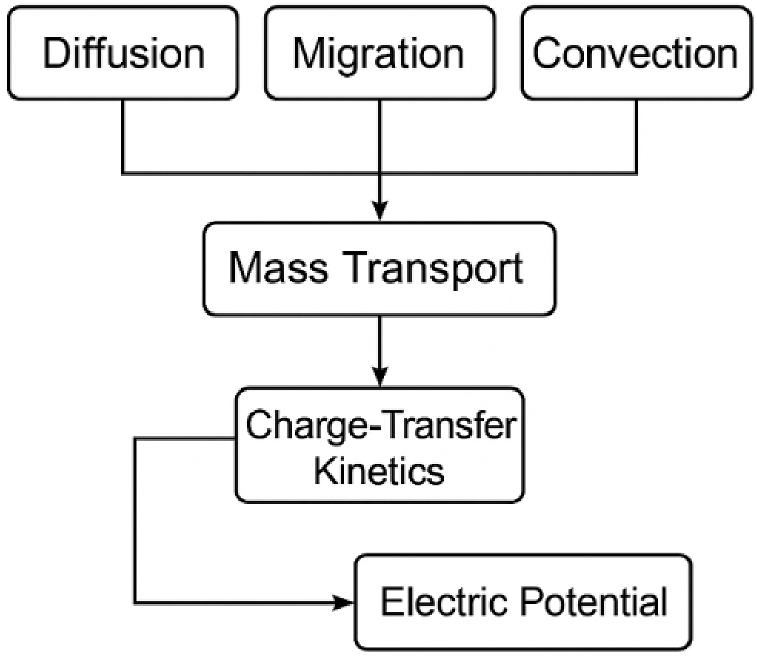

Figure S3: Simplified electrochemical system flowchart.

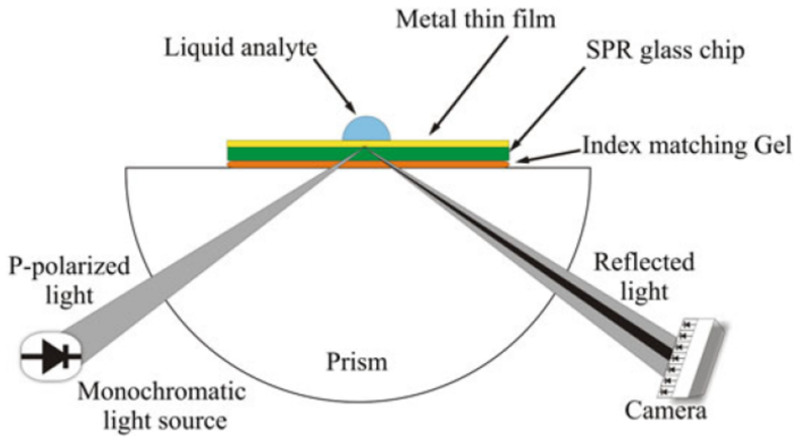

Figure S4: Schematic of SPR excitation in the Kretschmann configuration.

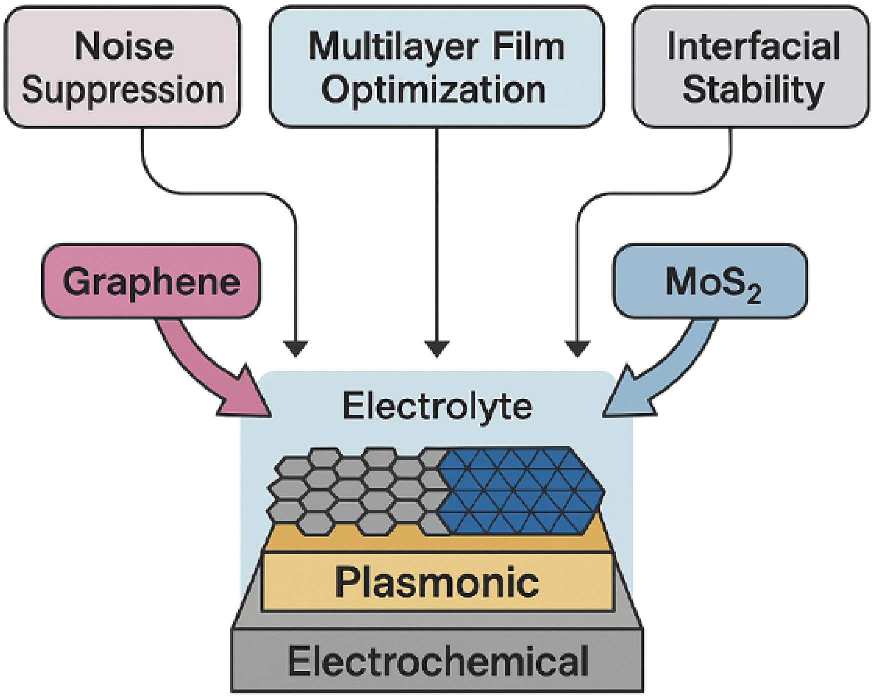

Figure S5: Technological challenges and nanomaterial integration in EC-SPR systems.

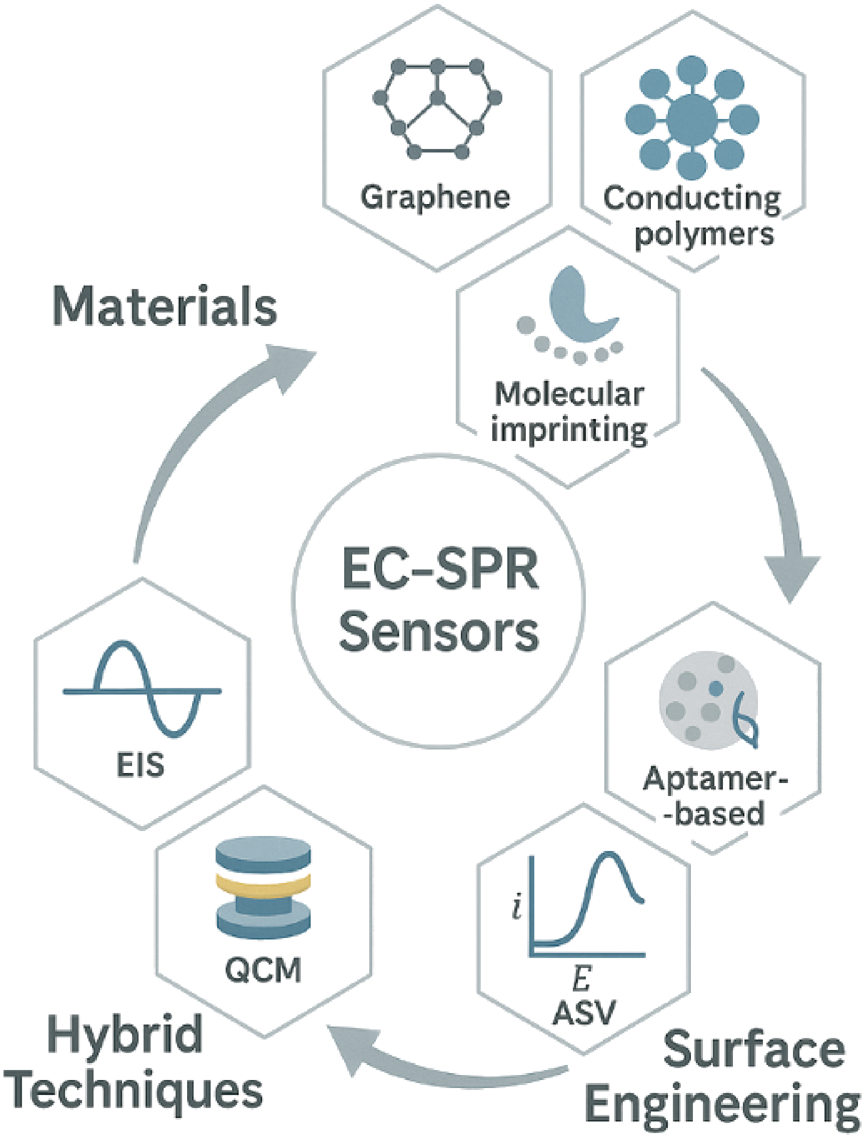

Figure S6: Strategies to overcome technological limitations in EC-SPR sensors.

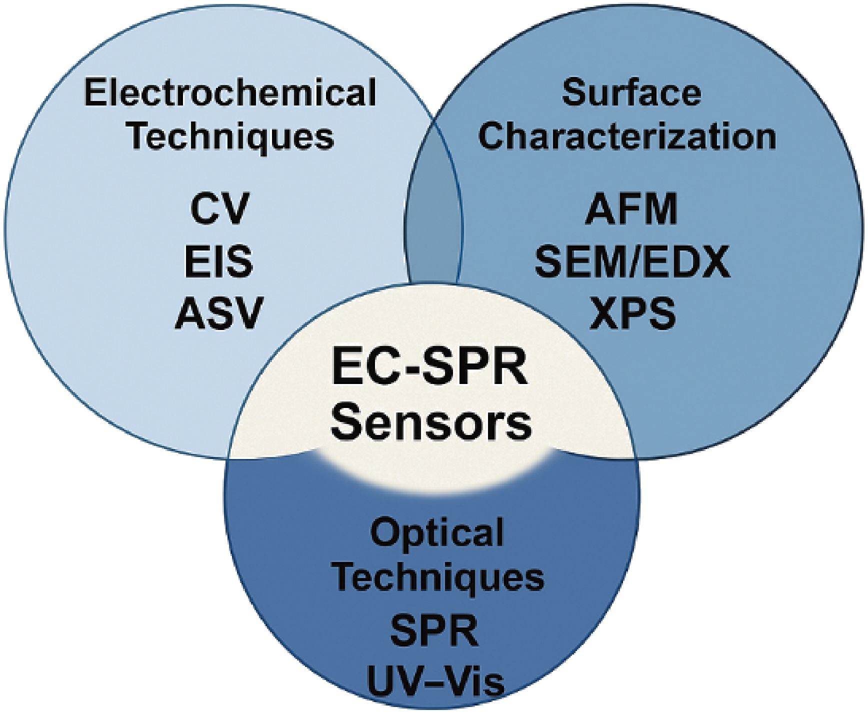

Figure S7: Strategies to overcome technological limitations in EC-SPR sensors.

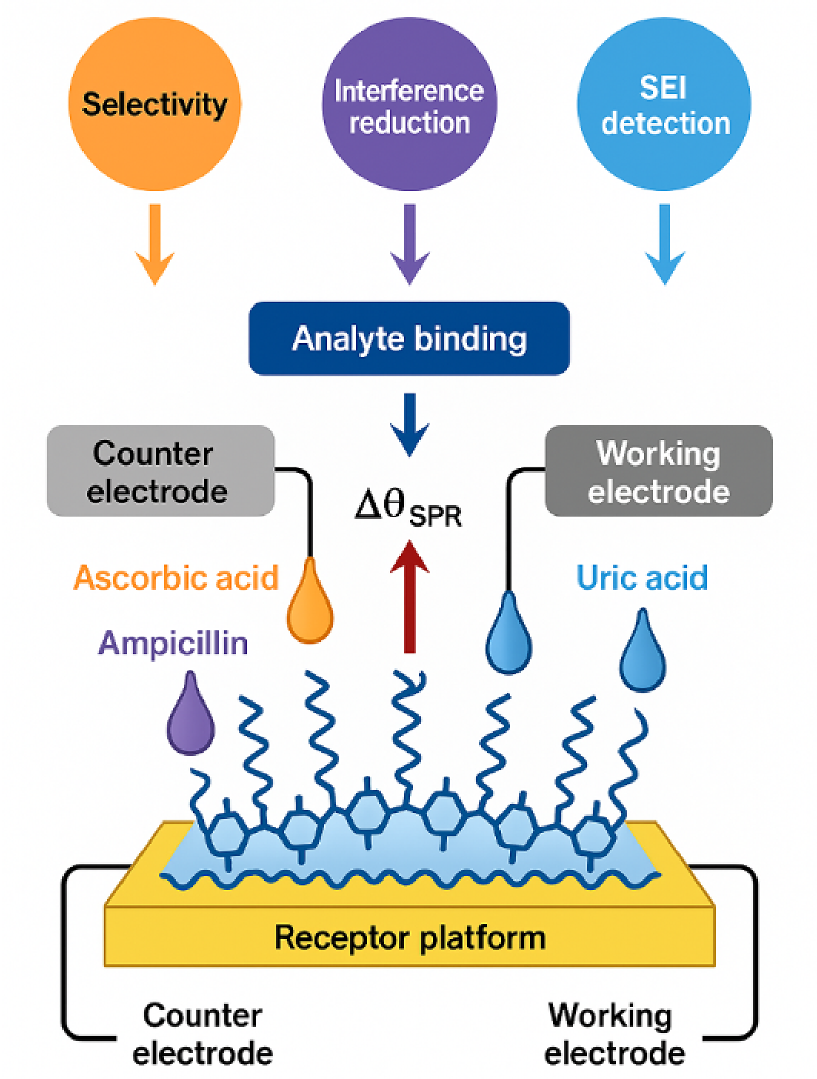

Figure S8: EC-SPR biosensing mechanisms and signal transduction in two-electrode systems.

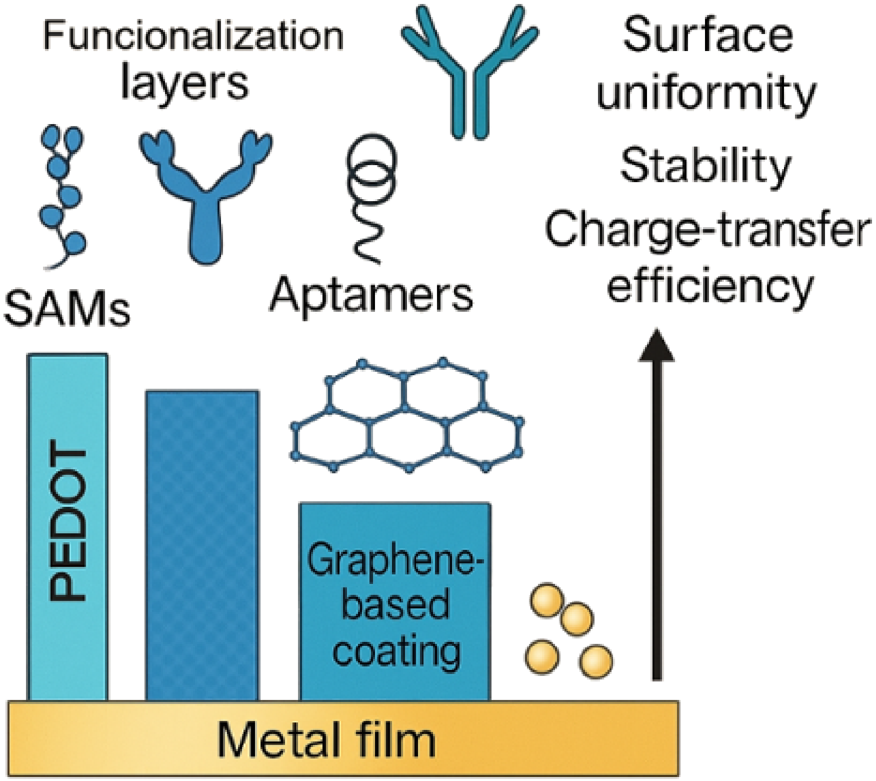

Figure S9: Material Architectures and Functional Layers in EC-SPR Sensors.

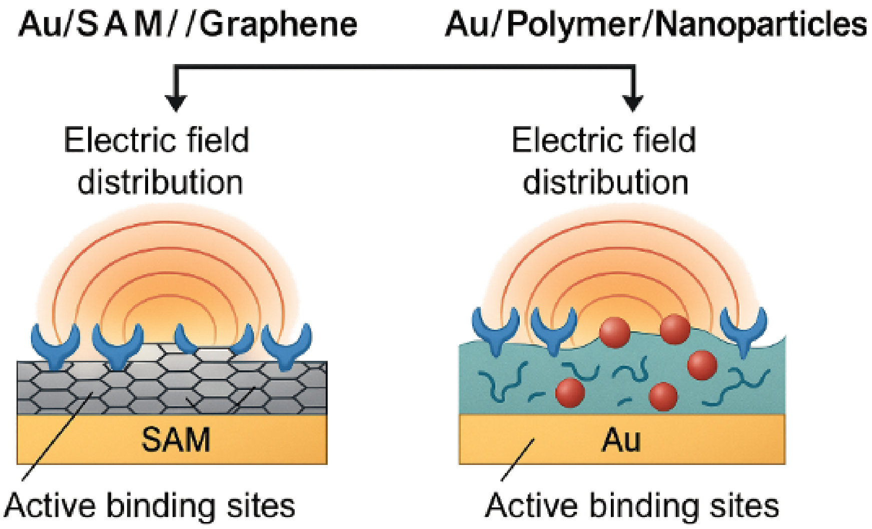

Figure S10: EC-SPR Chip Structures and Layer Configuration.

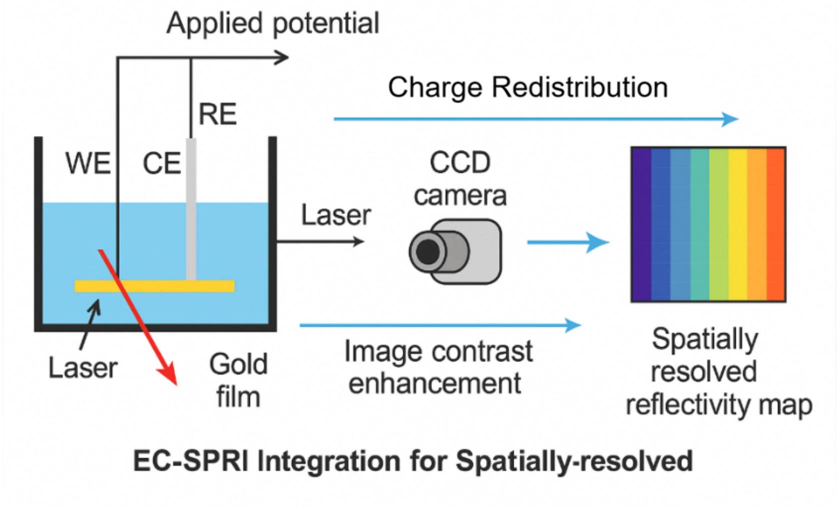

Figure S11: EC-SPRi Integration for Spatially Resolved Electro-Optical Mapping.

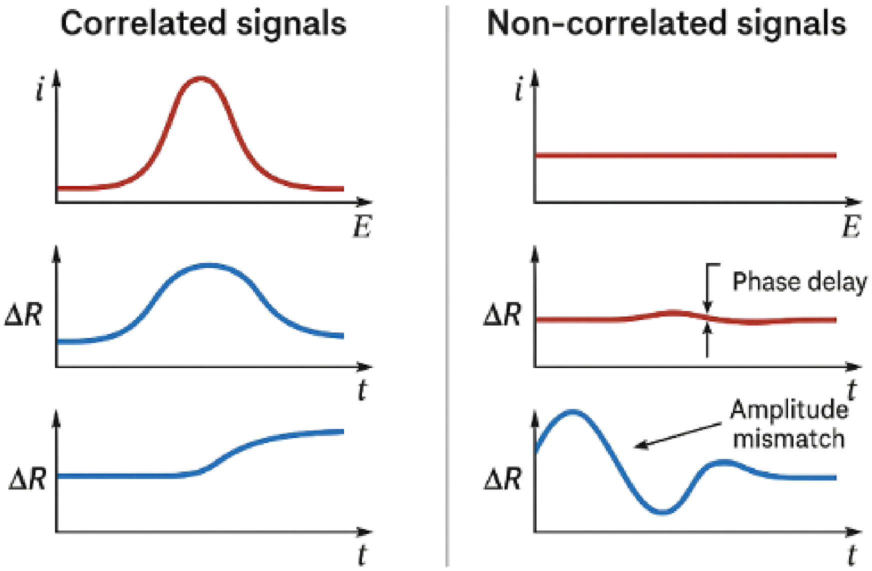

Figure S12: Comparison of Correlated and Non-Correlated EC-SPR Signal Responses.

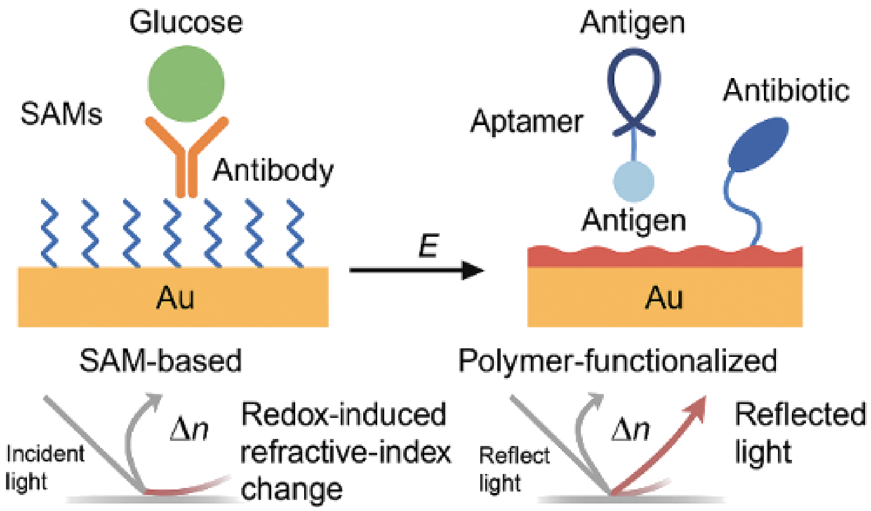

Figure S13: Biomolecule Immobilization Mechanisms in EC-SPR Biosensors.

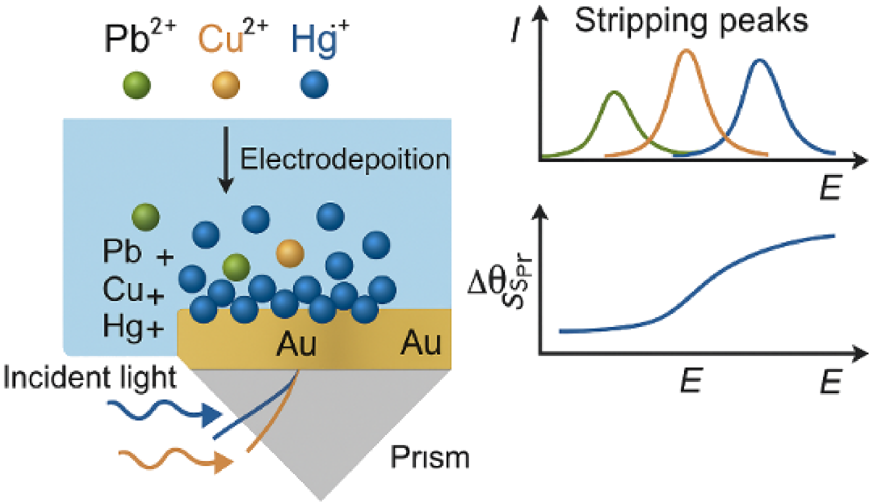

Figure S14: Integration of EC-SPR with ASV for Heavy Metal Detection.

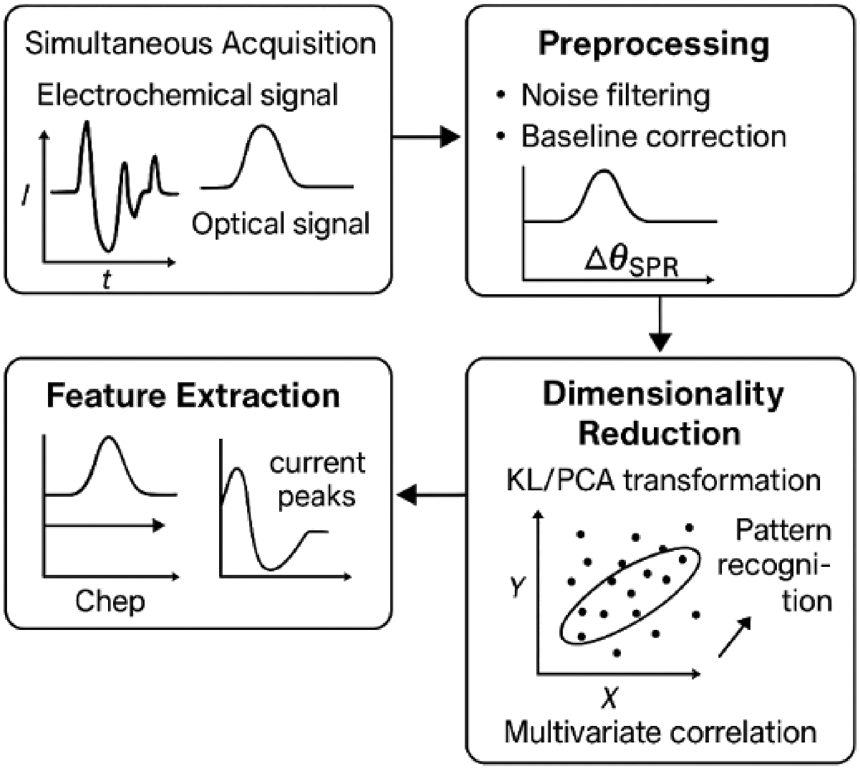

Figure S15: Data Processing Workflow in EC-SPR Analysis.

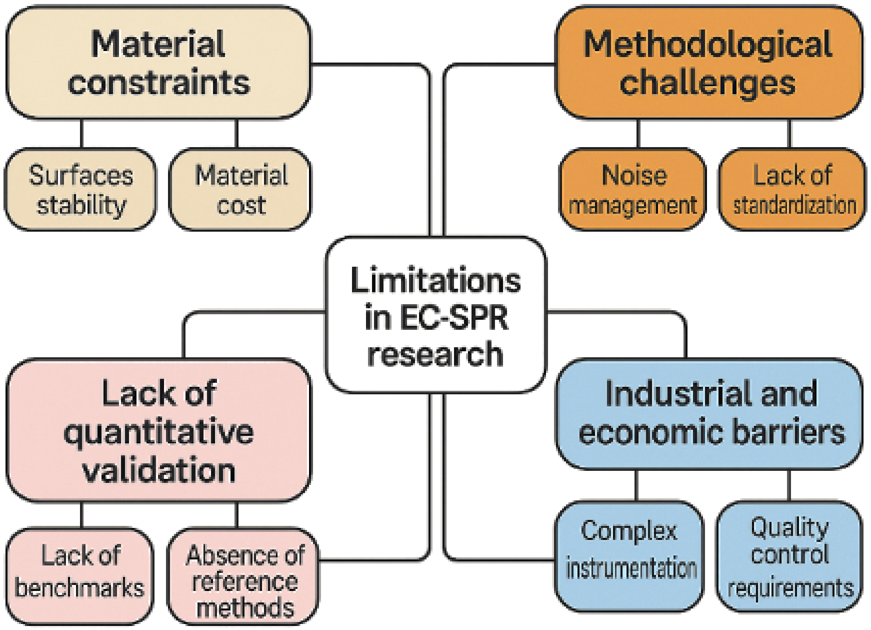

Figure S16: Conceptual Map of Weaknesses and Gaps in EC-SPR Research.

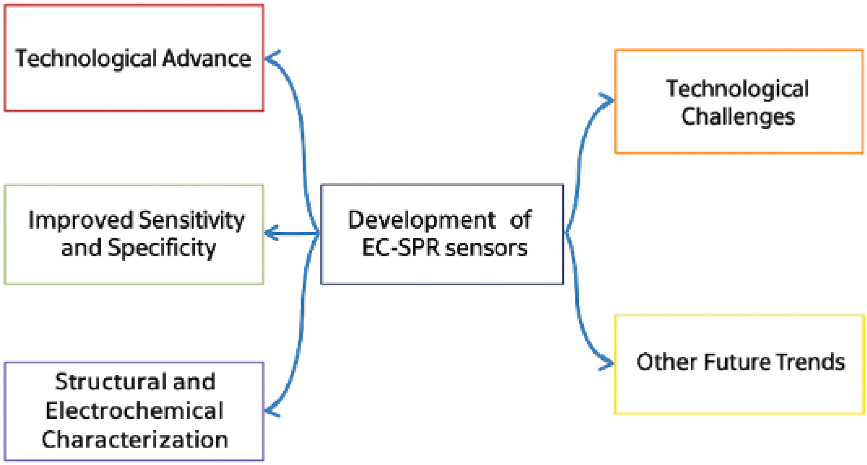

Figure S17: Conceptual Flowchart of EC-SPR Integration.

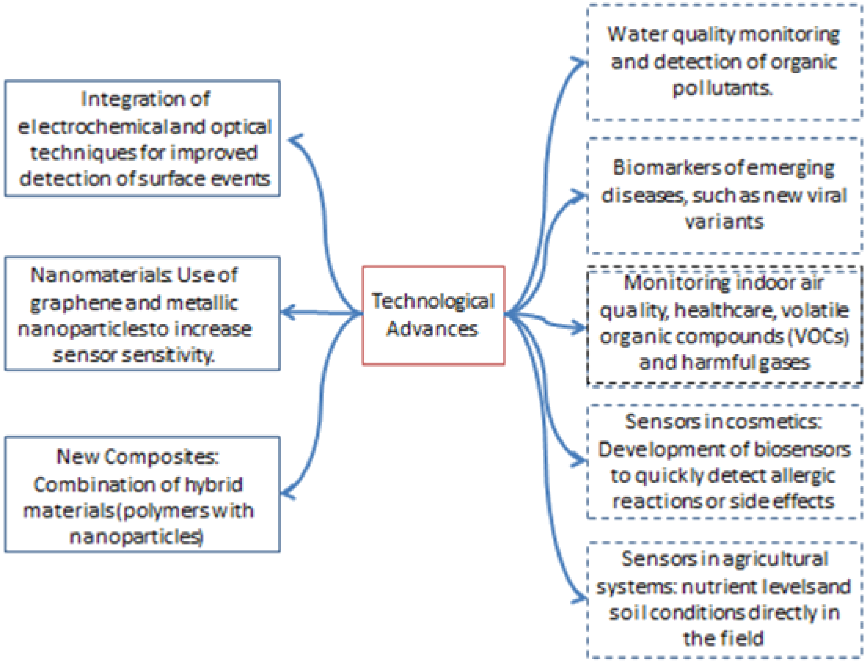

Figure S18: Technological Advances.

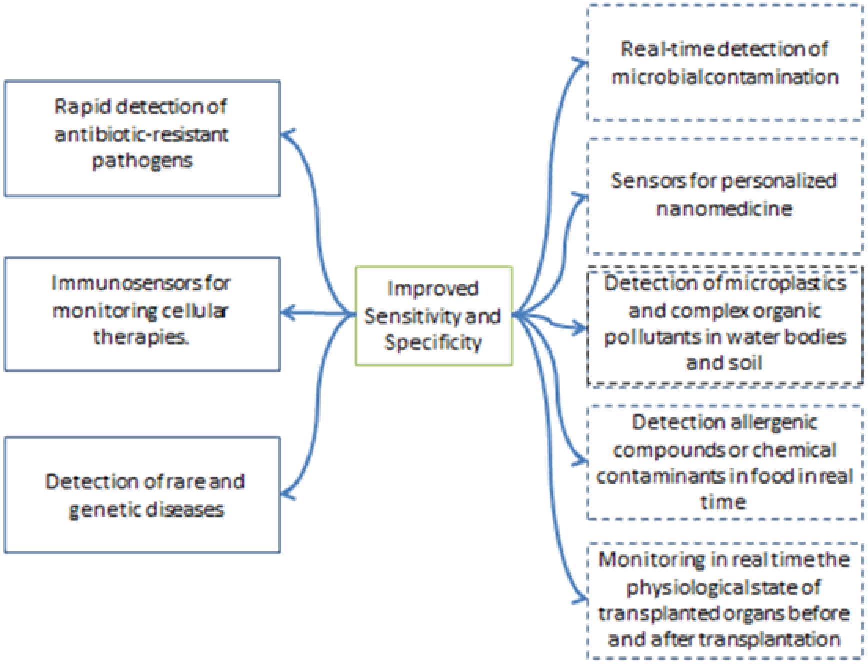

Figure S19: Strategic Applications Related to Improvements in EC-SPR Sensor Sensitivity and Specificity.

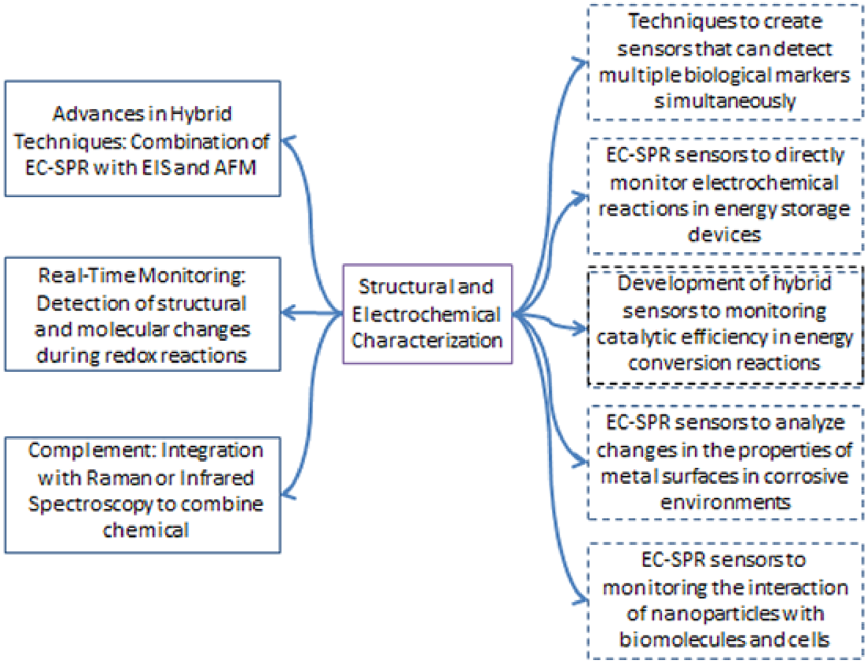

Figure S20: Advances in Structural and Electrochemical Characterization with EC-SPR Sensors and Their Multidisciplinary Applications.

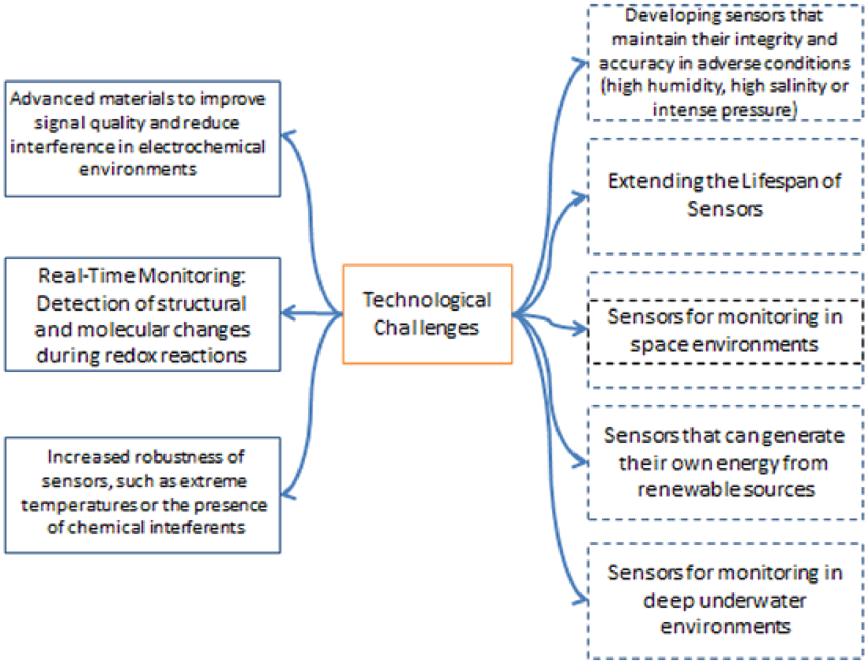

Figure S21: Main Challenges in Developing EC-SPR Sensors for Harsh Conditions.
